# Self-reported sleep duration and daytime napping are associated with renal hyperfiltration and microalbuminuria in an apparently healthy Chinese population

**DOI:** 10.1371/journal.pone.0214776

**Published:** 2019-08-30

**Authors:** Yingnan Ye, Linxi Zhang, Wenhua Yan, Anping Wang, Weiqing Wang, Zhengnan Gao, Xulei Tang, Li Yan, Qin Wan, Zuojie Luo, Guijun Qin, Lulu Chen, Shiqing Wang, Yuxia Wang, Yiming Mu

**Affiliations:** 1 Department of Endocrinology, Chinese PLA General Hospital, Beijing, China; 2 Department of Medicine, Nankai University, Tianjin, China; 3 Shanghai Jiaotong University Affiliated Ruijin Hospital, Shanghai, China; 4 Center Hospital of Dalian, Dalian, Liaoning, China; 5 Lanzhou University First Hospital, Lanzhou, Gansu, China; 6 Zhongshan University Sun Yat-sen Memorial Hospital, Guangzhou, Guangdong, China; 7 Southwest Medical University Affiliated Hospital, Luzhou, Sichuan, China; 8 Guangxi Medical University First Affiliated Hospital, Nanning, Guangxi, China; 9 Zhengzhou University First affiliated Hospital, Zhengzhou, Henan, China; 10 Wuhan Union Hospital, Wuhan, Hubei, China; University of Colorado Denver School of Medicine, UNITED STATES

## Abstract

**Background:**

Sleep duration affects health in various ways. The objective of the present study was to investigate the relationships among sleep duration, daytime napping and kidney function in a middle-aged apparently healthy Chinese population.

**Methods:**

According to self-reported total sleep and daytime napping durations, 33,850 participants who were 38–90 years old and recruited from eight regional centers were divided into subgroups. Height, weight, waist circumference, hip circumference, blood pressure, biochemical indexes, fasting blood glucose (FBG), postprandial blood glucose (PBG), HbA1c, creatinine and urinary albumin-creatinine ratio (UACR) were measured and recorded for each subject. Microalbuminuria was defined as UACR ≥30 mg/g, chronic kidney disease (CKD) was defined as eGFR <60 ml/min, and hyperfiltration was defined as eGFR ≥135 ml/min. Multiple logistic regression was applied to investigate the association between sleep and kidney function.

**Results:**

Compared to sleeping for 7–8 h/day, the ORs for microalbuminuria for sleeping for >9 h/day, 8–9 h/day 6–7 h/day and <6 h/day were 1.343 (1.228–1.470, *P*<0.001), 1.223 (1.134–1.320, *P*<0.001), 1.130 (1.003–1.273, *P* = 0.045) and 1.140 (0.908–1.431, *P* = 0.259), respectively. The eGFR levels exhibited a U-shaped association with sleep duration among subjects with an eGFR ≥90 ml/min and an N-shaped association with sleep duration among subjects with an eGFR <90 ml/min. The OR for hyperfiltration for >9 h/day of sleep was 1.400 (1.123–1.745, *P* = 0.003) among participants with an eGFR ≥90 ml/min. Daytime napping had a negative effect on renal health. Compared to the absence of a napping habit, the ORs for microalbuminuria for 0–1 h/day, 1–1.5 h/day and >1.5 h/day of daytime napping were 1.552 (1.444–1.668, *P*<0.001), 1.301 (1.135–1.491, *P*<0.001) and 1.567 (1.353–1.814, *P*<0.001), respectively.

**Conclusion:**

The association of total sleep duration with renal health outcomes is U-shaped. Daytime napping has a negative effect on renal health.

## Introduction

In recent decades, accumulating evidence has indicated that chronic sleep disorders represent a risk factor that affects metabolic health. Inappropriate sleep duration has been proven to be associated with many adverse health outcomes, such as diabetes [1, 2], obesity [3], hypertension [4, 5], osteoporosis [6], cardiovascular disease [7, 8], stroke [9] and total mortality [10]. Recently, a series of studies have suggested that extreme sleep durations may contribute to the decline in kidney function, which is closely correlated with the vascular system and is also an important and independent risk factor for cardiovascular disease. Both extremely short [11, 12] and long [13] sleep durations and poor sleep quality [14, 15] have been reported to be correlated with a higher urine albumin-to-creatinine ratio (UACR), which is a sensitive indicator for microalbuminuria or early-stage kidney damage, in a US and Japanese population. In addition, short sleep durations were associated with higher odds of inadequate hydration [16]. However, the determination of whether the effect of sleep duration on glomerular filtration rate (GFR) is positive or negative remains debatable. Several studies have demonstrated an increased risk of chronic kidney disease (CKD) [17, 18] or lower GFR [12, 19–22] in short sleepers, but a few studies have shown no correlation between sleep duration and CKD [23–26]. Conversely, both cross-sectional and cohort studies have reported that inappropriate sleep durations contribute to glomerular hyperfiltration [13, 15, 27, 28]. This difference in outcomes can be attributed not only to the differences in the race, age, social work stress, and health and economic status of the participants but also to the fact that during the progression of CKD, healthy individuals tend to initially have glomerular hyperfiltration, followed by increased risk for renal injury, leading to a decrease in filtration rate and an accelerated development of CKD [29–32]. This pathophysiological progression occurs in the context of type 1 diabetes and hypertension, as well as in the increasing stages of prediabetes and prehypertension [33–35]. Therefore, to provide further evidence for the contribution of sleep duration in the progression of renal function decline, multicenter studies with sufficient participants with diverse health conditions would be required. Therefore, the present study was conducted to determine whether the relationship between UACR and sleep duration exists among populations in Chinese provinces and cities and to verify whether different health conditions have an interactive effect on this relationship.

## Methods

### Ethics statement

The study protocol was approved by the Committee on Human Research at Rui-Jin Hospital affiliated with the School of Medicine, Shanghai Jiao Tong University. Written informed consents were obtained from all participants before data collection.

### Study subjects

A total of 33,850 participants from eight regional centers (Dalian, Guangzhou, Zhengzhou, Lanzhou, Luzhou, Wuhan, Guangxi and Shanghai) were included in the risk evaluation of cancers in Chinese diabetic individuals: a longitudinal (REACTION) study. Participants with primary kidney diseases and daily ACEI/ARB medicine use and those with a fallacious self-reported sleep duration (<4 hours or >12 hours) were excluded.

### Questionnaire

A standardized questionnaire was used to collect basic information, which included medical history, physical exercise, and smoking and alcohol consumption habits. Self-reported sleep duration and daytime napping duration were ascertained through the following questions: (1) “how many hours of sleep do you usually get at night?” and (2) “how many minutes do you usually spend napping in the afternoon?”. All investigators were previously trained. In the analysis, the participants were divided into four groups (<6, ≥6 and <7, ≥7 and ≤8, >8 and ≤9, and >9 h/day) based on their sleep duration, and 7–8 hours of sleep was generally considered the most appropriate sleep duration.

### Physical examination

The height, weight, waist circumference (WC) and hip circumference (HC) of these subjects were measured and recorded. The participants were asked to take off their shoes, hats and coats before the measurements. WC was measured at the horizontal midpoint between the anterior superior spine and the inferior margin of the 12^th^ rib. HC was defined as the horizontal length of the most protruding part of the hips. All data were recorded to within one decimal place.

### UACR measurement and data processing

Urine samples were collected in the morning for the UACR measurement. According to quartiles of the center to which the subjects belonged in the logistic regression, the UACR data were divided into the following groups: the under 25% group, the 25%-50% group, the 50%-75% group, and the over 75% group. Subjects with a higher UACR level were defined as those that belonged to the over 75% group. Microalbuminuria was defined as UACR ≥30 mg/g.

### Estimated GFR (eGFR) calculation

The Modification of Diet in Renal Disease (MDRD) formula was used to calculate the eGFR.

$$eGFR(ml/min\times 1.73m^{2})=186\times Scr{\left(mg/dl\right)}^{-1.154}\times age{\left(years\right)}^{-0.203}(female\times 0.742)$$

CKD was defined as eGFR <60 ml/min, while hyperfiltration was defined as eGFR ≥135 ml/min.

### Blood pressure measurement

Blood pressure was measured three times at one-minute intervals after the subjects were seated for five minutes. The average of the three values was used for the analysis. Hypertension was defined as an average systolic blood pressure ≥130 mmHg, a diastolic blood pressure ≥80 mmHg, or a definite medical history of hypertension.

### Blood biochemical index measurement

Blood samples were drawn in the morning after the subjects fasted for eight hours the previous night. Participants without a history of diabetes underwent a 75-g oral glucose tolerance test, while those with diabetes underwent a 100-g oral steamed bread tolerance test, and their venous blood samples were drawn at 0 and 120 minutes. The biochemical index included triglycerides (TG), total cholesterol (TC), low-density lipoprotein (LDL), high-density lipoprotein (HDL), creatinine (Scr), urea nitrogen (BUN), liver function indexes (ALT, AST, GGT), fasting blood glucose (FBG), postprandial blood glucose (PBG), glycosylated hemoglobin (HbA1c), fasting blood insulin and postprandial blood insulin, which were measured using the glucose oxidase-peroxidase method. Diabetes mellitus (DM) was defined as FBG ≥7.0 mmol/L, PBG ≥11.1 mmol/L, or a definite medical history of diabetes. Impaired fasting glucose was defined as FBG ≥6.1 mmol/L but without DM. Impaired glucose tolerance was defined as PBG ≥7.8 mmol/L but without DM.

### Statistical analysis

Statistical analysis was performed using SPSS software version 19.0 (Chicago, IL, USA). All continuous variables with a normal distribution are presented as the mean ± standard deviation (SD). All continuous variables with a skewed distribution are presented as the median and the 25^th^ and 75^th^ percentiles. All enumeration data are presented as proportions. The differences in the mean values or proportions of the characteristics of the studied subjects’ cross-sectional associations between sleep duration and UACR, FPG, PPG, AST, GGT, ALT, HbA1c and TG values were determined, and the latter values were natural log transformed in the analyses due to their skewed distribution. ANOVA (for measurement data) and chi-square (for enumeration data) tests were performed to test the significant differences in the general characteristics between the groups. Multivariate logistic regression was used to calculate the odds ratio values (OR values) for various endpoint outcomes based on the different sleep or daytime napping duration groups. Groups with the lowest OR values were set as the reference. In the adjusted model, confounding factors were also incorporated into the regression equations. Among these confounding factors, exercise, smoking and alcohol consumption habits were classified into different grades according to the results of the questionnaire survey, and these were included in the equation as classified variables, together with sex. The remaining confounding factors, which included age, Hip and waist circumference, BMI, TG, TC, HDL, LDL, GGT, blood glucose and blood pressure, were included in the equation as continuous variables with or without logarithmic conversion. A joint analysis performed by combining the categories of sleep duration with daytime napping duration in the regression model was conducted to analyze the interaction between daytime napping and sleep durations using subjects with no napping habits and 7–8 hours of sleep duration as the reference. Finally, to explore the effects of diverse indicators on the relationship between sleep and renal function, these participants were divided into subgroups based on blood pressure, blood glucose and BMI, and regression models were established for each subgroup.

## Results

A total of 33,850 participants, comprising 11,198 males and 22,652 females, were included in the analysis. The mean age of the total population was 58.1 ± 9.3 years old, the self-reported total sleep duration was 8.1 ± 1.2 hours, the daytime napping duration was 0.3 ± 0.6 hours, the median UACR was 10.0 mg/g, and the mean eGFR was 93.9 ± 19.1 ml/min.

According to the different categories of total sleep duration, the general characteristics of the participants in the present study are presented in Table 1. Compared with those who slept for 7–8 hours per day, participants who slept for shorter or longer durations were more likely to be older and have higher TG, PBG and UACR values, as well as a higher prevalence of diabetes, hypertension, hyperfiltration and microalbuminuria, and a high UACR level. Those with sleep durations longer than 9 hours were more likely to be obese and have lower eGFR levels and perform less physical exercise. However, their TC and LDL levels were lower than the reference. Opposite results were observed in the short sleep duration groups. Furthermore, no significant difference in blood pressure was observed across all sleep categories. Although there were significant differences in alcohol consumption and smoking habits across the groups, no significant increase or decrease or U-shaped relationship between sleep duration and alcohol consumption or smoking was observed.

**Table 1:** Baseline information according to self-reported total sleep duration.

Sleep duration, h	ALL n = 33850	(0, 6) n = 651	[6, 7) n = 2854	[7, 8] n = 16492	(8, 9] n = 8902	>9 n = 4951	P value
**Age, years**	58.1±9.3	58.7±8.8	57.7±8.8	57.5±8.8	58.5±9.6	59.8±10.3	<0.001
**Male, %**	33.1(11198)	31.5(205)	31.7(904)	32.1(5291)	33.7(2997)	36.4(1801)	<0.001
**Waist circumference, cm**	86.2±10.0	87.6±11.2	86.4±10.1	86.0±9.9	86.3±10.0	86.4±10.2	<0.001
**Hip circumference, cm**	97.0±7.8	97.7±8.6	97.3±7.7	97.0±7.8	97.0±7.8	96.6±7.9	<0.001
**BMI, kg/m2**	24.7±3.7	25.1±4.3	24.9±3.6	24.7±3.7	24.6±3.6	24.5±3.7	<0.001
**Tumor, %**	2.9(992)	3.7(24)	3.3(93)	2.9(485)	2.8(252)	2.8(138)	0.515
**Diabetes, %**	22.8(7712)	24.7(161)	21.8(622)	21.0(3461)	23.4(2082)	28.0(1386)	<0.001
**Smoking habits**							<0.001
**Regular smoker, %**	12.6(4219)	13.2(85)	13.2(372)	12.1(1983)	12.2(1075)	14.3(704)	
**Sometimes smoker, %**	2.6(878)	1.4(9)	2.4(68)	2.5(407)	2.9(257)	2.8(137)	
**Never smoker, %**	84.8(28470)	85.4(551)	83.5(2384)	85.4(13975)	84.9(7493)	82.9(4067)	
**Drinking habits**							<0.001
**Regular drinker, %**	7.0(2349)	9.6(62)	7.7(219)	6.6(1080)	7.1(626)	7.4(362)	
**Sometimes drinker, %**	19.1(6406)	17.0(110)	19.8(560)	19.9(3262)	17.9(1580)	18.2(894)	
**Never drinker, %**	73.9(24827)	73.4(475)	72.4(2048)	73.5(12034)	75.0(6620)	74.4(3650)	
**Regular exercise, %**	12.1(4097)	14.0(91)	12.9(368)	12.9(2133)	11.3(1008)	9.9(497)	<0.001
**Exercise intensity**							<0.001
**Mild exercise, %**	9.1(3081)	9.8(64)	9.7(278)	10.2(1675)	8.4(750)	6.3(314)	
**Moderate exercise, %**	2.6(875)	3.4(22)	2.8(80)	2.4(403)	2.5(224)	2.9(146)	
**Severe exercise, %**	0.4(141)	0.8(5)	0.4(10)	0.3(55)	0.4(34)	0.7(37)	
**Obesity(BMI> = 28), %**	15.1(5127)	18.9(123)	17.4(2854)	15.2(2510)	14.5(1293)	14.2(703)	<0.001
**HbA1c, %**	5.9(5.6,6.2)	5.9(5.6,6.3)	5.9(5.6,6.2)	5.9(5.6,6.2)	5.9(5.6,6.3)	5.9(5.6,6.4)	<0.001
**SBP, mmHg**	131.8±20.4	132.5±22.0	132.1±20.2	131.9±20.3	131.6±20.4	131.9±20.8	0.695
**DBP, mmHh**	77.6±10.9	77.4±11.1	77.6±11.0	77.6±10.9	77.5±10.8	77.6±10.9	0.795
**Hypertension, %**	60.3(20403)	62.1(404)	60.8(1734)	60.0(9898)	60.3(5364)	60.7(3003)	<0.001
**CreaC, mmol/L**	65.8(59.5,73.7)	65.3(59.1,72.6)	65.2(59.1,73.2)	65.4(59.3,73.1)	65.9(59.5,74.2)	67.0(60.3,75.8)	<0.001
**Triglycerides, mmol/L**	1.36(0.97,1.97)	1.36(0.98,1.96)	1.32(0.92,1.92)	1.32(0.95,1.92)	1.41(1.00,2.02)	1.44(1.02,2.07)	<0.001
**Cholesterol, mmol/L**	5.07±1.13	5.16±1.14	5.13±1.12	5.12±1.13	5.01±1.13	4.95±1.13	<0.001
**HDL, mmol/L**	1.32±0.34	1.33±0.37	1.34±0.34	1.33±0.34	1.30±0.33	1.28±0.34	<0.001
**LDL, mmol/L**	2.98±0.90	3.03±0.91	3.04±0.89	3.02±0.89	2.94±0.90	2.88±0.89	<0.001
**GGT, mmol/L**	21.0(15.0,32.0)	22.0(16.0,33.0)	21.0(15.0,32.0)	21.0(15.0,32.0)	21.0(15.0,33.0)	21.0(15.0,33.0)	0.400
**AST, mmol/L**	20.0(17.0,25.0)	20.0(17.0,25.0)	20.0(17.0,25.0)	20.0(17.0,25.0)	20.0(17.0,25.0)	20.0(17.0,25.0)	0.921
**ALT, mmol/L**	15.0(11.0,21.0)	15.0(11.0,21.0)	15.0(11.0,21.0)	15.0(11.0,21.0)	15.0(11.0,21.0)	15.0(11.0,21.0)	0.234
**FBG, mmol/L**	5.55(5.12,6.19)	5.58(5.14,6.30)	5.56(5.12,6.17)	5.53(5.12,6.11)	5.55(5.12,6.20)	5.60(5.14,6.36)	<0.001
**PBG, mmol/L**	7.40(6.01,9.73)	7.54(6.10,10.10)	7.35(5.91,9.62)	7.26(5.97,9.47)	7.48(6.08,9.82)	7.79(6.19,10.56)	<0.001
**Daytime napping, %**	33.7(11393)	10.6(69)	12.5(358)	18.9(3117)	48.4(4307)	71.5(3542)	<0.001
**Microalbuminuria, %**	14.5(4905)	15.2(99)	14.2(404)	12.6(2079)	15.9(1411)	18.4(912)	<0.001
**High UACR level, %**	24.7(8357)	26.7(174)	26.0(741)	23.8(3920)	24.6(2189)	26.9(1333)	<0.001
**CKD, %**	2.5(832)	2.6(17)	2.0(56)	2.1(354)	2.5(226)	3.6(179)	<0.001
**Hyperfiltration, %**	2.5(860)	2.8(18)	2.3(65)	2.2(363)	2.8(250)	3.3(164)	<0.001
**UACR, mg/g**	10.0(5.8,19.9)	9.7(5.8,19.7)	9.4(5.7,18.2)	9.0(5.4,17.9)	10.9(6.1,21.7)	12.3(6.7,24.0)	<0.001
**eGFR, ml/(min×1.73m**^**2**^	93.9±19.1	94.4±19.7	94.3±18.6	94.2±18.4	93.9±19.5	92.9±20.6	<0.001

Chi-squared tests for discrete variables and one-way ANOVA for continuous variables.

All continuous variables with normal distribution are presented as the mean values and standard deviation (SD). All continuous variables with skewness distribution are presented as media and 25,75 percentile. All enumeration data presented as proportion.

Microalbuminuria was defined as UACR> = 30 mg/g, CKD was defined as eGFR<60 ml/min and hyperfiltration was defined as eGFR> = 135 ml/min.

According to the quartile division, among which centre the subjects belonged, the UACR data were divided into the under 25% group, the 25%-50% group, the 50%-75% group, and the over 75% group. Higher UACR level was defined as subjects belonged to the over 75% group.

Table 2 summarizes the characteristics of participants by daytime napping duration. Napping habits were reported by 7,001 of 11,198 males (62.5%) and 15,456 of 22,652 females (68.2%). Those who took naps appeared to be more likely to be female, which is contrary to the results of a previous study [27]. Individuals who took naps were more likely to be regular alcohol consumers and smokers and to perform less physical exercise. Naturally, these individuals also had higher BMI and TG values and were more likely to have metabolic diseases, such as obesity, hypertension, and especially diabetes. Interestingly, napping appeared to be a protective factor for hypercholesterolemia due to the negative association between nap duration and CHOL and HDL. This result is consistent with the relationship between total sleep duration and CHOL and HDL. Napping habits also contribute to kidney function decline. Participants who took naps had a higher prevalence of microalbuminuria, CKD and hyperfiltration, and their UACR values were significantly higher.

**Table 2:** Baseline information according to self-reported daytime napping.

Daytime napping, h	ALL n = 33850	0 n = 22457	(0, 1] n = 8240	(1, 1.5] n = 1817	>1.5 n = 1336	P value
**Age, years**	58.1±9.3	57.3±8.9	59.8±9.7	59.7±9.6	59.5±9.8	<0.001
**Male, %**	33.1(11198)	31.2(7001)	35.9(2960)	41.8(759)	35.8(478)	<0.001
**Waist circumference, cm**	86.2±10.0	86.2±10.1	86.0±9.8	86.7±10.1	87.3±10.4	<0.001
**Hip circumference, cm**	97.0±7.8	97.0±7.9	96.8±7.6	97.2±7.8	97.4±7.8	0.042
**BMI, kg/m2**	24.7±3.7	24.7±3.7	24.4±3.6	24.8±3.8	25.1±3.8	<0.001
**Tumor, %**	2.9(992)	3.1(698)	2.6(212)	3.2(58)	1.8(24)	0.004
**Diabetes, %**	22.8(7712)	20.7(4657)	25.3(2084)	29.8(541)	32.2(430)	<0.001
**Smoking habits**						<0.001
**Regular smoker, %**	12.6(4219)	12.3(2745)	12.1(996)	16.3(291)	14.2(187)	
**Sometimes smoker, %**	2.6(878)	2.5(553)	2.9(236)	2.9(52)	2.8(37)	
**Never smoker, %**	84.8(28470)	85.2(18961)	85.0(6969)	80.8(1443)	83.0(1097)	
**Drinking habits**						<0.001
**Regular drinker, %**	7.0(2349)	6.8(1507)	6.8(559)	9.8(175)	8.1(108)	
**Sometimes drinker, %**	19.1(6406)	19.1(4242)	18.9(1554)	20.3(363)	18.6(247)	
**Never drinker, %**	73.9(24827)	73.5(16512)	74.3(6095)	69.9(1247)	73.3(973)	
**Regular exercise, %**	12.1(4097)	13.3(2972)	9.7(802)	9.5(173)	11.2(150)	<0.001
**Exercise intensity**						<0.001
**Mild exercise, %**	9.1(3081)	10.0(2251)	7.5(620)	5.8(106)	7.8(104)	
**Moderate exercise, %**	2.6(875)	2.8(618)	1.9(158)	3.4(61)	2.8(38)	
**Severe exercise, %**	0.4(141)	0.5(103)	0.3(24)	0.3(6)	0.6(8)	
**Obesity(BMI> = 28), %**	15.1(5127)	15.7(3515)	13.3(1092)	15.9(289)	17.3(231)	<0.001
**HbA1c, %**	5.9(5.6,6.2)	5.8(5.6,6.2)	5.9(5.6,6.3)	6.0(5.6,6.4)	6.0(5.6,6.5)	<0.001
**SBP, mmHg**	131.8±20.4	132.1±20.6	131.4±20.0	131.6±20.2	131.1±19.7	0.034
**DBP, mmHg**	77.6±10.9	77.8±11.0	77.1±10.6	77.9±10.7	77.3±10.8	<0.001
**Hypertension, %**	60.3(20403)	60.2(13530)	59.8(4926)	62.6(1137)	60.6(810)	<0.001
**CreaC, mmol/L**	65.8(59.5,73.7)	65.2(59.1,72.9)	66.9(50.4,75.3)	66.9(60.1,77.8)	66.4(60.0,74.2)	<0.001
**Triglycerides, mmol/L**	1.36(0.97,1.97)	1.32(0.95,1.92)	1.45(1.03,2.06)	1.43(1.03,2.08)	1.49(1.04,2.10)	<0.001
**Cholesterol, mmol/L**	5.07±1.13	5.16±1.13	4.88±1.11	4.95±1.16	4.85±1.10	<0.001
**HDL, mmol/L**	1.32±0.34	1.34±0.34	1.26±0.33	1.26±0.34	1.25±0.33	<0.001
**LDL, mmol/L**	2.98±0.90	3.06±0.90	2.83±0.87	2.87±0.88	2.79±0.85	<0.001
**GGT, mmol/L**	21.0(15.0,32.0)	21.0(15.0,32.0)	20.0(15.0,31.8)	22.0(15.0,34.0)	21.0(15.0,33.0)	<0.001
**AST, mmol/L**	20.0(17.0,25.0)	20.0(17.0,25.0)	20.0(17.0,25.0)	20.0(17.0,25.0)	21.0(17.0,25.0)	0.862
**ALT, mmol/L**	15.0(11.0,21.0)	15.0(11.0,21.0)	15.0(11.0,21.0)	14.0(11.0,20.0)	15.0(11.0,21.0)	0.715
**FBG, mmol/L**	5.55(5.12,6.19)	5.52(5.12,6.11)	5.57(5.10,6.26)	5.65(5.20,6.40)	5.70(5.21,6.50)	<0.001
**PBG, mmol/L**	7.40(6.01,9.73)	7.24(5.95,9.44)	7.62(6.10,10.10)	8.00(6.46,10.89)	8.23(6.50,11.10)	<0.001
**Total sleep duration, h**	8.1±1.2	7.8±1.1	8.5±1.0	9.2±1.1	9.8±1.2	<0.001
**Microalbuminuria, %**	14.5(4905)	12.2(2742)	19.3(1589)	16.8(305)	20.1(269)	<0.001
**High UACR level, %**	24.7(8357)	24.2(5434)	24.8(2045)	27.5(500)	28.3(378)	<0.001
**CKD, %**	2.5(832)	2.3(510)	2.6(216)	3.6(65)	3.1(41)	0.001
**Hyperfilitration, %**	2.5(860)	2.3(517)	2.7(226)	3.7(68)	3.7(49)	<0.001
**UACR, mg/g**	10.0(5.8,19.9)	8.7(5.3,16.9)	13.7(7.7,25.3)	11.3(6.2,22.0)	12.8(7.1,26.5)	<0.001
**eGFR, ml/(min×1.73m**^**2**^	93.9±19.1	94.3±18.5	94.3±18.6	93.8±21.2	93.8±20.2	<0.001

Chi-squared tests for discrete variables and one-way ANOVA for continuous variables.

All continuous variables with normal distribution are presented as the mean values and standard deviation (SD). All continuous variables with skewness distribution are presented as media and 25,75 percentile. All enumeration data presented as proportion.

Microalbuminuria was defined as UACR> = 30 mg/g, CKD was defined as eGFR<60 ml/min and hyperfiltration was defined as eGFR> = 135 ml/min.

According to the quartile division, among which centre the subjects belonged, the UACR data were divided into the under 25% group, the 25%-50% group, the 50%-75% group, and the over 75% group. Higher UACR level was defined as subjects belonged to the over 75% group.

**Fig 1:**
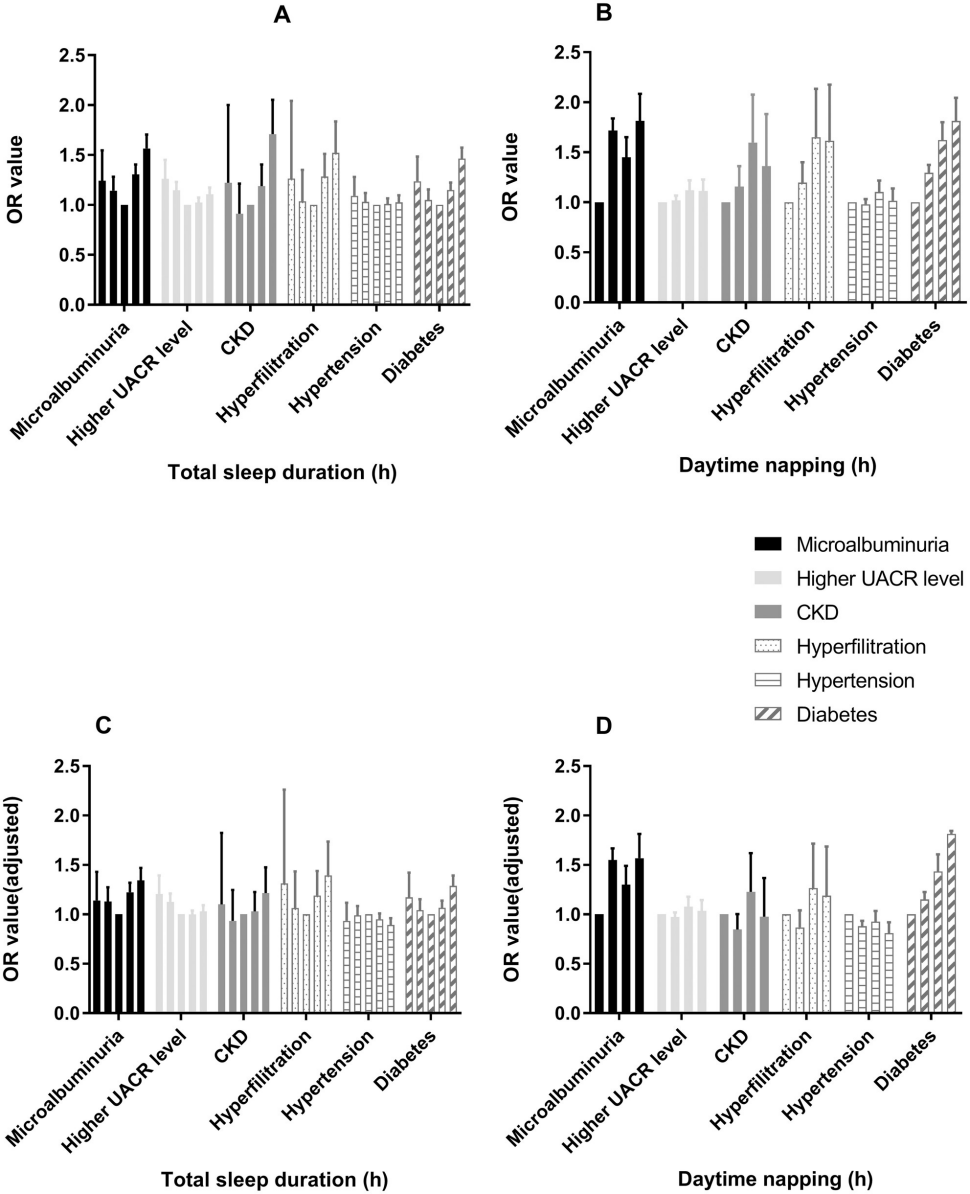
The association between sleep duration and risks for multiple health outcomes. (A and C) The U-shaped association between total sleep duration and risks for multiple health outcomes. (B and D) The positive association between daytime napping duration and risks for multiple health outcomes. The bars in each group of data in the figure represent the total sleep duration of <6, 6–7, 7–8, 8–9 and >9 hours or represent the daytime napping duration of 0, 0–1, 1–1.5 and >1.5 hours, successively. Figs 1C and 1D were adjusted for age, sex, BMI, HC, WC, alcohol consumption and smoking habits, physical exercise, TG, TC, HDL, LDL, GGT, blood glucose (except for diabetes), and blood pressure (except for hypertension).

The association between sleep duration and several health outcomes, including microalbuminuria, high UACR level, CKD, hyperfiltration, hypertension and diabetes, was an additional focus. Multivariate logistic regressions were carried out before and after adjustment for possible confounding variables, such as age, sex, BMI, alcohol consumption and smoking habits, physical exercise, TG, CHOL, FBG, PBG, HbA1c, systolic blood pressure (SBP), diastolic blood pressure (DBP), HDL, LDL, WC and HC. The results are presented in Table 3. The participants reporting short or long sleep duration had significantly higher risks for microalbuminuria, high UACR level and diabetes before adjustment in model 1, suggesting a U-shaped relationship between sleep duration and health outcomes (Fig 1A).

**Table 3:** Risk for health outcomes according to total sleep duration and daytime napping.

	microalbuminuria		higher UACR level		CKD		hyperfiltration		hypertension		diabetes	
	OR	P value	OR	P value	OR	P value	OR	P value	OR	P value	OR	P value
**Model 1**												
**Total sleep duration,h**												
**(0, 6)**	1.243(0.999, 1.547)	0.051	1.262(1.097, 1.452)	0.001	1.222(0.747, 2.001)	0.425	1.263(0.782, 2.042)	0.339	1.090(0.927, 1.280)	0.297	1.237(1.031, 1.484)	0.022
**[6, 7)**	1.143(1.019, 1.282)	0.022	1.147(1.068, 1.231)	<0.001	0.912(0.686, 1.213)	0.528	1.036(0.793, 1.352)	0.798	1.031(0.951, 1.119)	0.456	1.049(0.953, 1.155)	0.329
**[7, 8]**	1		1		1		1		1		1	
**(8, 9]**	1.306(1.214, 1.405)	<0.001	1.025(0.978, 1.074)	0.296	1.188(1.003, 1.406)	0.046	1.284(1.091, 1.511)	0.003	1.010(0.958, 1.065)	0.710	1.149(1.081, 1.223)	<0.001
**>9**	1.565(1.437, 1.705)	<0.001	1.108(1.047, 1.174)	<0.001	1.710(1.425, 2.053)	<0.001	1.522(1.262, 1.836)	<0.001	1.027(0.962, 1.096)	0.422	1.464(1.361, 1.574)	<0.001
**Daytime napping,h**												
**0**	1		1		1		1		1		1	
**(0, 1]**	1.718(1.605, 1.838)	<0.001	1.019(0.974, 1.067)	0.398	1.158(0.986, 1.361)	0.074	1.197(1.021, 1.402)	0.026	0.981(0.931, 1.033)	0.459	1.294(1.220, 1.373)	<0.001
**(1, 1.5]**	1.450(1.274, 1.651)	<0.001	1.120(1.028, 1.220)	0.009	1.597(1.228, 2.076)	<0.001	1.650(1.275, 2.134)	<0.001	1.103(1.000, 1.218)	0.051	1.621(1.458, 1.801)	<0.001
**>1.5**	1.813(1.577, 2.084)	<0.001	1.113(1.008, 1.229)	0.034	1.362(0.986, 1.882)	0.061	1.616(1.199, 2.177)	0.002	1.016(0.908, 1.138)	0.783	1.814(1.610, 2.044)	<0.001
**Model 2**												
**Total sleep duration,h**												
**(0, 6)**	1.167(0.935, 1.456)	0.172	1.212(1.051, 1.395)	0.008	1.065(0.645, 1.759)	0.806	1.346(0.829, 2.184)	0.229	0.967(0.813, 1.149)	0.700	1.159(0.961, 1.398)	0.122
**[6, 7)**	1.127(1.003, 1.266)	0.044	1.129(1.050, 1.212)	0.001	0.887(0.665, 1.184)	0.416	1.043(0.797, 1.365)	0.758	0.987(0.904, 1.077)	0.767	1.029(0.932, 1.136)	0.570
**[7, 8]**	1		1		1		1		1		1	
**(8, 9]**	1.262(1.172, 1.359)	<0.001	1.002(0.957, 1.050)	0.927	1.031(0.868, 1.224)	0.731	1.324(1.123, 1.561)	0.001	0.971(0.917, 1.027)	0.303	1.104(1.036, 1.177)	0.002
**>9**	1.448(1.327, 1.579)	<0.001	1.064(1.004, 1.126)	0.035	1.273(1.054, 1.538)	0.012	1.636(1.354, 1.977)	<0.001	0.921(0.858, 0.989)	0.023	1.341(1.244, 1.446)	<0.001
**Daytime napping,h**												
**0**	1		1		1		1		1		1	
**(0, 1]**	1.602(1.495, 1.716)	<0.001	0.973(0.930, 1.019)	0.244	0.887(0.752, 1.047)	0.157	1.304(1.110, 1.530)	0.001	0.863(0.816, 0.913)	<0.001	1.180(1.110, 1.255)	<0.001
**(1, 1.5]**	1.368(1.200, 1.560)	<0.001	1.112(1.020, 1.212)	0.016	1.295(0.990, 1.693)	0.059	1.731(1.333, 2.247)	<0.001	0.926(0.832, 1.030)	0.158	1.427(1.279, 1.592)	<0.001
**>1.5**	1.674(1.453, 1.929)	<0.001	1.053(0.954, 1.164)	0.305	1.055(0.759, 1.468)	0.749	1.761(1.302, 2.382)	<0.001	0.822(0.727, 0.929)	0.002	1.619(1.431, 1.831)	<0.001
**Model 3**												
**Total sleep duration,h**												
**(0, 6)**	1.140(0.908, 1.431)	0.259	1.207(1.045, 1.392)	0.010	1.100(0.663, 1.824)	0.713	1.314(0.763, 2.261)	0.325	0.935(0.782, 1.118)	0.461	1.171(0.965, 1.421)	0.110
**[6, 7)**	1.130(1.003, 1.273)	0.045	1.126(1.048, 1.212)	0.001	0.934(0.699, 1.247)	0.642	1.064(0.789, 1.435)	0.683	0.991(0.905, 1.085)	0.844	1.042(0.940, 1.154)	0.436
**[7, 8]**	1		1		1		1		1		1	
**(8, 9]**	1.223(1.134, 1.320)	<0.001	0.990(0.945, 1.039)	0.688	1.030(0.866, 1.226)	0.736	1.191(0.987, 1.438)	0.068	0.951(0.897, 1.009)	0.096	1.065(0.997, 1.138)	0.060
**>9**	1.343(1.228, 1.470)	<0.001	1.030(0.971, 1.092)	0.322	1.216(1.002, 1.477)	0.047	1.395(1.121, 1.736)	0.003	0.893(0.830, 0.961)	0.003	1.288(1.191, 1.393)	<0.001
**Daytime napping,h**												
**0**	1		1		1		1		1		1	
**(0, 1]**	1.552(1.444, 1.668)	<0.001	0.973(0.930, 1.020)	0.267	0.847(0.715, 1.003)	0.054	0.867(0.721, 1.041)	0.126	0.881(0.831, 0.934)	<0.001	1.150(1.079, 1.226)	<0.001
**(1, 1.5]**	1.301(1.135, 1.491)	<0.001	1.079(0.988, 1.179)	0.091	1.229(0.933, 1.619)	0.143	1.265(0.933, 1.716)	0.130	0.926(0.828, 1.035)	0.175	1.434(1.279, 1.608)	<0.001
**>1.5**	1.567(1.353, 1.814)	<0.001	1.035(0.934, 1.145)	0.517	0.976(0.696, 1.367)	0.886	1.188(0.837, 1.687)	0.336	0.809(0.713, 0.919)	0.001	1.623(1.427, 1.845)	<0.001

Model 1 was unadjusted. Model 2 was adjusted for age, sex and BMI. Model 3 was adjusted for covariates in model 2 plus hip and waist circumference, drinking and smoking habits, physical exercise, triglycerides, cholesterol, HDL, LDL, GGT, blood glucose (except for diabetes) and blood pressure (except for hypertension).

After adjusting for age, sex and BMI in model 2, the significance of the relationship between short sleep duration and diabetes decreased. After all confounding variables were adjusted, only the following data were statistically significant. Compared with the reference, the fully adjusted ORs for >9 h/day of sleep for microalbuminuria and diabetes were 1.343 (1.228–1.470, *P*<0.001) and 1.288 (1.191–1.393, *P*<0.001), respectively; the OR for 8–9 h/day of sleep for microalbuminuria was 1.223 (1.134–1.320, *P*<0.001); the ORs for <6 h/day and 6–7 h/day of sleep for a high UACR level were 1.207 (1.045–1.392, *P* = 0.010) and 1.126 (1.048–1.212, *P* = 0.001), respectively; and the OR for <6–7 h/day of sleep for microalbuminuria was 1.130 (1.003–1.273, *P* = 0.045). Sleep duration had little effect on blood pressure levels. Only after adjusting for all confounding variables, >9 hours of sleep appeared to have a slight effect on blood pressure (OR = 0.893, *P* = 0.001). Therefore, these confounders have a strong interaction in the relationship between sleep duration and urinary protein, but the U-shaped trend relationship between sleep duration and urinary protein existed and was independent of these confounders (Fig 1C).

According to the daytime napping duration categories, it was found that daytime napping duration is a risk factor for microalbuminuria and diabetes, which was independent of the confounders. Compared to subjects who did not nap, the ORs of individuals who napped for 0–1 h/day, 1–1.5 h/day and >1.5 h/day for microalbuminuria were 1.552 (1.444–1.668, *P*<0.001), 1.301 (1.135–1.491, *P*<0.001) and 1.567 (1.353–1.814, *P*<0.001), respectively, while for diabetes, these ORs were 1.150 (1.079–1.226, *P*<0.001), 1.434 (1.279–1.608, *P*<0.001) and 1.623 (1.427–1.845, *P*<0.001), respectively, after full adjustments (Fig 1B and 1D).

**Fig 2:**
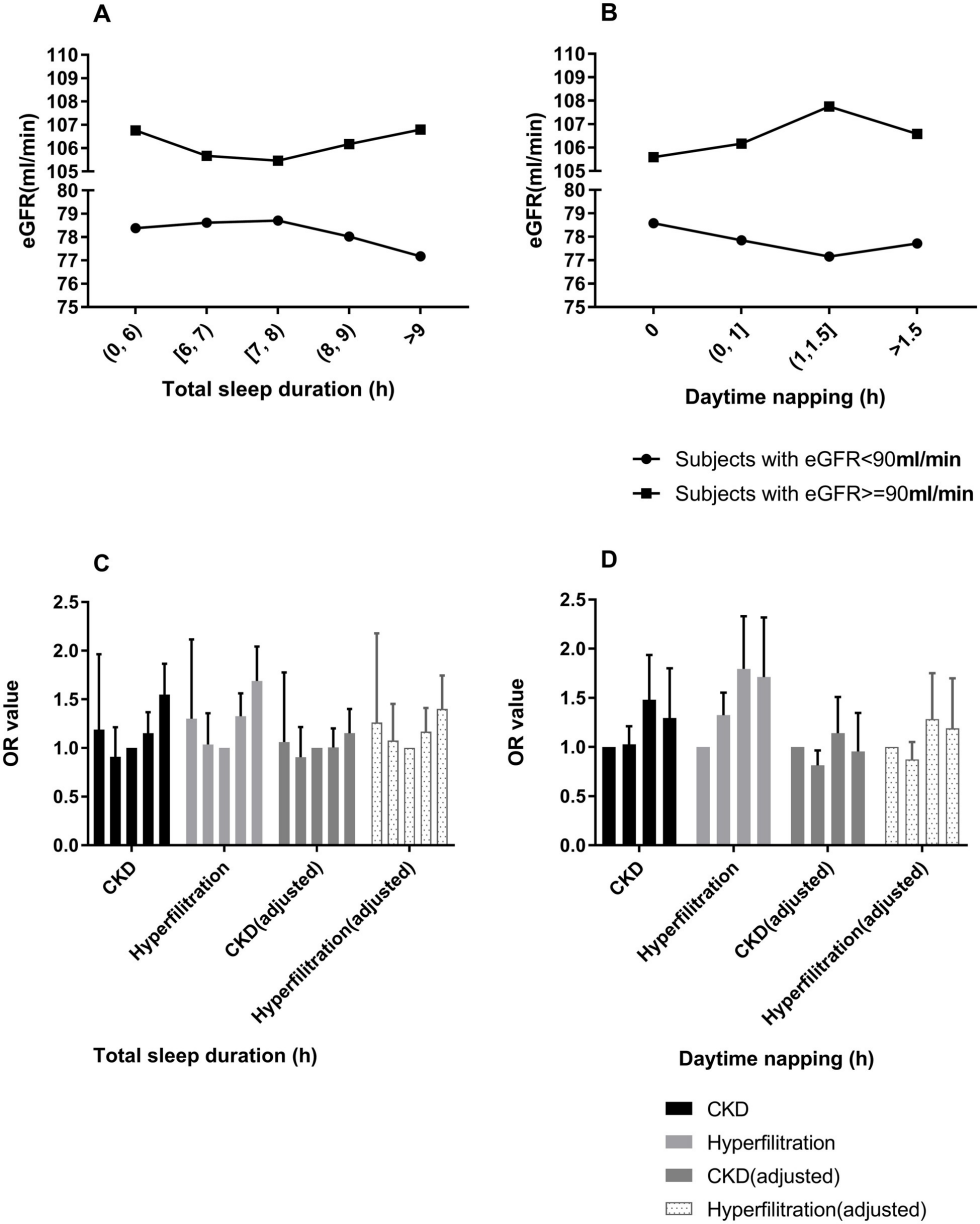
The association between sleep duration and eGFR. (A) The association between mean eGFR values and total sleep duration among subjects with <90 ml/min and ≥90 ml/min eGFR. (B) The association between mean eGFR values and daytime napping duration among subjects with <90 ml/min and ≥90 ml/min of eGFR. (C and D) Risks for hyperfiltration among subjects with ≥90 ml/min eGFR and risks for CKD among subjects with <90 ml/min eGFR, according to total sleep duration or daytime napping duration. The adjusted covariates included age, sex, BMI, HC, WC, alcohol consumption and smoking habits, physical exercise, TG, TC, HDL, LDL, GGT, blood glucose and blood pressure. CKD was defined as eGFR <60 ml/min, and hyperfiltration was defined as eGFR ≥135 ml/min.

To further investigate the relationship between total or daytime sleep duration and eGFR, CKD and hyperfiltration, the participants were divided into two groups: eGFR ≥90 ml/min group and eGFR <90 ml/min group. In the progression of CKD, healthy individuals tended to initially have glomerular hyperfiltration, followed by an increased risk for renal injury, leading to a decrease in filtration rate and the acceleration of the development of CKD [29–32]. Thus, it was speculated that the eGFR level is positively associated with inappropriate sleep duration among subjects with normal eGFR but negatively associated with inappropriate sleep duration among subjects with lower eGFR. These results confirm the present conjecture. The eGFR value exhibited a U-shaped association with sleep duration in the normal eGFR group (Fig 2A) and an N-shaped associated with sleep duration in the lower eGFR group (Fig 2B), suggesting that both short and long sleep duration contributes to the progression of kidney function decline, and this finding was consistent with the urinary protein-sleep duration relationship. Multivariate logistic regressions were carried out to examine the results (Table 4) after full adjustments, the OR for >9 h/day of sleep for hyperfiltration was 1.400 (1.123–1.745, *P* = 0.003) in the eGFR ≥90 group. The U-shaped trend relationships of sleep duration with hyperfiltration for normal eGFR subjects or of CKD for lower eGFR subjects existed (Fig 2C), although the statistical significance was not strong. The reason for this lack of strong significance could be because few of the present participants had an eGFR of <90 ml/min.

**Table 4:** Relationships between total sleep duration, daytime napping and eGFR.

	CKD risk for subjects with eGFR<90		Hyperfiltration risk for subjects with eGFR> = 90	
	OR for CKD	P value	OR for hyperfiltration	P value
**Model 1**				
**Total sleep duration,h**				
**(0, 6)**	1.188(0.720, 1.963)	0.500	1.302(0.802, 2.116)	0.286
**[6, 7)**	0.910(0.681, 1.214)	0.520	1.036(0.792, 1.357)	0.794
**[7, 8]**	1		1	
**(8, 9]**	1.152(0.971, 1.368)	0.105	1.325(1.123, 1.562)	0.001
**>9**	1.549(1.286, 1.866)	<0.001	1.689(1.397, 2.042)	<0.001
**Daytime napping,h**				
**0**	1		1	
**(0, 1]**	1.029(0.873, 1.212)	0.736	1.324(1.128, 1.554)	0.001
**(1, 1.5]**	1.481(1.132, 1.937)	0.004	1.794(1.381, 2.330)	<0.001
**>1.5**	1.294(0.931, 1.800)	0.125	1.712(1.265, 2.317)	<0.001
**Model 2**				
**Total sleep duration,h**				
**(0, 6)**	1.053(0.633, 1.752)	0.843	1.336(0.820, 2.176)	0.245
**[6, 7)**	0.863(0.644, 1.156)	0.322	1.037(0.791, 1.360)	0.791
**[7, 8]**	1		1	
**(8, 9]**	1.006(0.844, 1.198)	0.950	1.326(1.123, 1.565)	0.001
**>9**	1.208(0.997, 1.464)	0.053	1.688(1.394, 2.044)	<0.001
**Daytime napping,h**				
**0**			1	
**(0, 1]**	0.860(0.727, 1.017)	0.077	1.345(1.144, 1.581)	<0.001
**(1, 1.5]**	1.212(0.921, 1.594)	0.170	1.793(1.377, 2.334)	<0.001
**>1.5**	1.064(0.761, 1.488)	0.718	1.736(1.279, 2.357)	<0.001
**Model 3**				
**Total sleep duration,h**				
**(0, 6)**	1.062(0.635, 1.777)	0.818	1.261(0.729, 2.179)	0.407
**[6, 7)**	0.906(0.676, 1.216)	0.512	1.076(0.797, 1.453)	0.633
**[7, 8]**	1		1	
**(8, 9]**	1.005(0.842, 1.200)	0.957	1.168(0.966, 1.411)	0.109
**>9**	1.150(0.945, 1.401)	0.163	1.400(1.123, 1.745)	0.003
**Daytime napping,h**				
**0**	1		1	
**(0, 1]**	0.813(0.685, 0.965)	0.018	0.874(0.726, 1.051)	0.153
**(1, 1.5]**	1.140(0.860, 1.510)	0.363	1.285(0.943, 1.750)	0.112
**>1.5**	0.955(0.678, 1.347)	0.795	1.191(0.836, 1.699)	0.333

Model 1 was unadjusted. Model 2 was adjusted for age, sex and BMI. Model 3 was adjusted for covariates in model 2 plus hip and waist circumference, drinking and smoking habits, physical exercise, triglycerides, cholesterol, HDL, LDL, GGT, blood glucose and blood pressure.

It was observed that daytime napping had a positive effect on the risk for hyperfiltration in the eGFR ≥90 ml/min group in models 1 and 2 (Table 4, Fig 2D). However, after full adjustment, the statistical significance disappeared, suggesting that daytime napping affects the occurrence of hyperfiltration by influencing confounding factors, such as blood glucose, BMI and blood lipids. This idea can be supported by the previous findings of the investigators, in which napping significantly increased the risk of diabetes (Table 3).

**Fig 3:**
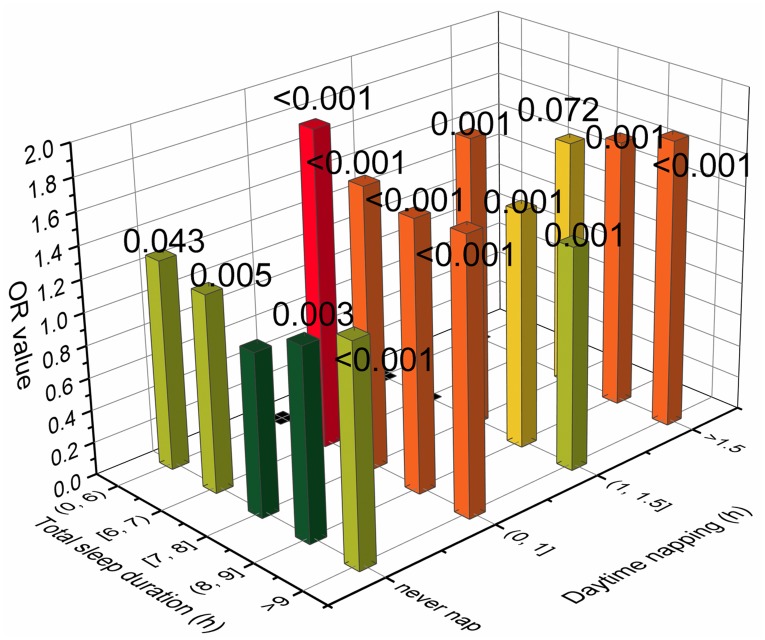
Joint analysis of total sleep duration and daytime napping duration in relation to the risk for microalbuminuria. The multivariate ORs for microalbuminuria were adjusted for age, sex, BMI, HC, WC, alcohol consumption and smoking habits, physical exercise, TG, TC, HDL, LDL, GGT, blood glucose and blood pressure. The asterisk denotes that the result was significantly different from 7–8 hours of sleep duration per day without daytime napping.

In fact, total sleep duration and daytime napping duration also had an interaction effect on the risk of health outcomes. Subjects who sleep for a short time usually do not nap, while those who sleep for a long time often have a napping habit. Therefore, a joint analysis was conducted to investigate this interaction. The participants were divided into 20 subgroups according to their total sleep duration and daytime napping duration. The following five groups were excluded from the reanalysis because there were fewer than 100 people: total sleep duration <6 hours and daytime napping between 0 and 1 hours; total sleep duration <6 hours and daytime napping between 1 and 1.5 hours; total sleep duration <6 hours and daytime napping >1.5 hours; total sleep duration between 6 and 7 hours and daytime napping between 1 and 1.5 hours; and total sleep duration between 6 and 7 hours and daytime napping >1.5 hours. A multivariate logistic regression after full adjustment was carried out, and the results are presented in Fig 3. Several of the results were notable. First, subjects with 7–8 hours of total sleep duration and did not have a napping habit had the lowest risk for microalbuminuria. Second, the U-shaped relationship of risk for microalbuminuria and sleep duration was significant in the nonnapping group, and the ORs of all these groups reached a statistically significant level (*P* = 0.043, 0.005, 0.003 and <0.001, respectively). This result not only indicates that the U-shaped curve relationship is more convincing but also explains why the statistical significance of short sleep duration groups was poor in the previous logistic regression analysis. The reason for this was because subjects with short total sleep durations often do not have a napping habit, and napping is also a risk factor for microalbuminuria, which prevents them from having a significantly higher risk for microalbuminuria when compared to the reference. Third, subjects with 6–7 hours of total sleep duration and 0–1 hours of daytime napping had the highest risk for microalbuminuria (OR = 1.945, *P*<0.001). This group had the shortest total sleep duration and the longest napping duration, and this was included in the analysis (groups with shorter total sleep duration and longer napping were excluded due to the small sample size, and the *P*-values of these groups were poor). This finding suggests that lack of nighttime sleep can be dangerous for people who are already short on total sleep duration, and daytime napping is not enough to make up for the loss of sleep at night, which is consistent with the conjecture of Miao *et al*. [27]. Fourth, subjects with the longest total sleep duration and the longest napping duration had the second highest risk for albuminuria (OR = 1.762, *P*<0.001), suggesting that longer napping duration would further aggravate the risk of extremely long total sleep duration and its effect on microalbuminuria.

Last, the interactions of several confounders were investigated, including blood pressure, blood glucose and BMI, which have been commonly considered to be the main risk factors for microalbuminuria. These results are presented in Table 5. It was found that long sleep duration was a statistically significant independent risk factor for microalbuminuria among subjects with SBP ≥110 mmHg and DBP <90 mmHg but not among subjects with SBP <110 mmHg or DBP ≥90 mmHg. This finding may be because blood pressure is the main factor that affects urinary protein, and the effect of sleep duration on urinary protein is nonsignificant for subjects with extremely poor or excellent blood pressure control. According to the results of different categories of glucose metabolism and BMI, both blood glucose and BMI interact with the sleep-albuminuria association. For subjects with abnormal glucose metabolism or obesity problems, poor sleep habits were even worse for their health. This finding could be explained by the above findings of Tables 1 and 3, in which extremely long sleep durations aggravated obesity or diabetes, followed by the worse effects that long sleep duration cause for their health, and this becomes a vicious cycle.

**Table 5:** Risk for microalbuminuria according to total sleep duration among diverse subgroups.

	Total sleep duration, h				
	(0, 6)	[6–7)	[7–8]	(8–9]	>9
**SBP(mmHg)**					
<110	0.791(0.592)	1.103(0.643)	1	1.198(0.168)	1.160(0.352)
110–120	1.794(0.025)	0.958(0.816)	1	1.335(0.005)	1.456(0.002)
120–130	0.853(0.619)	1.082(0.586)	1	1.174(0.087)	1.259(0.040)
≥130	1.095(0.550)	1.192(0.023)	1	1.207(<0.001)	1.361(<0.001)
**DBP(mmHg)**					
<80	1.504(0.005)	1.146(0.102)	1	1.248(<0.001)	1.461(<0.001)
80–90	0.720(0.209)	1.146(0.238)	1	1.203(0.013)	1.297(0.003)
≥90	0.761(0.360)	1.047(0.743)	1	1.185(0.064)	1.077(0.506)
**glycometabolism**					
NGT	1.225(0.267)	1.171(0.091)	1	1.171(0.009)	1.248(0.003)
IFG/IGT	1.083(0.717)	1.034(0.770)	1	1.280(0.001)	1.276(0.005)
DM	1.083(0.700)	1.157(0.200)	1	1.225(0.005)	1.489(<0.001)
**BMI(kg/m**^**2**^**)**					
<24	0.828(0.364)	1.038(0.707)	1	1.203(0.002)	1.329(<0.001)
24–27	1.104(0.636)	1.255(0.031)	1	1.205(0.007)	1.382(<0.001)
27–30	1.742(0.023)	1.182(0.226)	1	1.310(0.004)	1.264(0.037)
≥30	1.261(0.474)	0.996(0.983)	1	1.212(0.158)	1.418(0.033)

The models were adjusted for age, sex, BMI, hip and waist circumference, drinking and smoking habits, physical exercise, triglycerides, cholesterol, HDL, LDL, GGT, blood glucose and blood pressure. The data are presented as OR(P values).

## Discussion

The present study identified the associations between total sleep duration, daytime napping, and the incidence of microalbuminuria, high UACR levels, CKD and hyperfiltration in a community of middle-aged Chinese subjects. It was found that total sleep duration exhibited a U-shaped association with the incidence of microalbuminuria and that the progression of kidney function decline and daytime napping was positively associated with the incidence of microalbuminuria.

It was speculated that extremely long sleep durations could significantly aggravate the process of kidney damage, which initially leads to hyperfiltration and subsequently leads to eGFR decline. Further joint analysis revealed the importance of sleep duration on kidney function outcomes, in which people with short sleep durations should ensure enough nighttime sleep, while people with long sleep durations should limit daytime napping.

The lack of a strong association between blood pressure and sleep duration shows that blood pressure and kidney function are closely correlated. There are two possible explanations for this. First, the salt intake of Asians, especially the Chinese population, seriously exceeds the recommended limit; their sodium intake often exceeds 200 mmol/day, which is more than twice the global average level, while the daily physiological sodium requirement is only 10–20 mmol [36]. This makes sodium intake a major contributor to blood pressure levels in the Chinese population. However, the effect of sleep regulation independent of salt and sodium restriction on blood pressure was not enough to produce significant result. Second, blood pressure is affected by many factors, including obesity, mood, and hormones. All of these factors can be the cause or result of inappropriate sleep duration. This makes the relationship between sleep and blood pressure particularly complex, and it was speculated that the effects of sleep on blood pressure are often offset by the effects of sleep on those factors. For example, sleep deprivation often raises blood pressure, and people who do not get enough sleep tend to be thinner. However, lower body weight is a protective factor for hypertension. This may also explain the fact that in the present study, after adjusting for confounders, there was a statistically significant association between excessively long sleep duration and blood pressure. The present data suggest that sleeping >9 hours has a slight effect on lowering blood pressure. A meta-analysis also reported that >9 hours of sleep resulted in an abnormal decrease in the blood pressure-sleep duration curve when only nighttime sleep was considered or when the analysis was restricted to a population of only males [37]. However, this kind of blood pressure regulation is not sufficient to offset the negative effects of long sleep durations on kidney function. In fact, the effect of >9 hours sleep on blood pressure lost its statistical significance when an attempt was made to determine the hypertension outcome based on the old hypertension diagnostic criteria (exceeding 140/90 mmHg, data not shown). Therefore, it was speculated that sleep control has a very limited antihypertensive function, which does not effectively reduce the risk of systolic blood pressure exceeding 140 mmHg or effectively prevent hypertension complications.

Several studies have investigated the association between sleep and kidney health outcomes. Both short and long sleep durations were reported to be associated with decreased eGFR and the progression to end-stage renal disease (ESRD) in CKD [12, 22, 38, 39] or hypertension [19] populations and were associated with increased eGFR, hyperfiltration, inadequate hydration and the prevalence of CKD in a community-based general population [13, 15, 20, 21, 27, 28]. In the present study, based on the same general community population, it was further demonstrated that inappropriate sleep durations had negative effects on eGFR in a healthy or early-stage nephropathy population, which was consistent with the results of previous studies. However, a US study of 4,238 participants from the Nurses’ Health Study (NHS) reported that short sleep duration was prospectively associated with faster decline in kidney function in a healthy general population [11]. An explanation for this finding is that the NHS study was based on subjects who were nurses, who are often shift workers with highly intense work. A Japanese study suggested that inappropriate sleep duration was more likely to affect kidney health and raise the risk of early-stage kidney disease in shift workers [26]. Indeed, the participants of the NHS study had a lower mean eGFR (88.3 ± 25.0) than the present participants (93.9 ± 19.1). Therefore, their results were more similar to those in the CKD population. Few studies have selected albuminuria as a terminal outcome to evaluate how sleep affects kidney function. Both short and long sleep durations have been reported to be associated with UACR levels in populations from Japan [40], Korea [41] and the US [12], and daytime napping has been reported to be positively associated with albuminuria in Japan [42]. However, this relationship is race-specific [13], and it remains unknown whether this relationship exists in the Chinese population. The present study confirms the U-shaped relationship between sleep duration and albuminuria and the positive relationship between napping duration and albuminuria in the Chinese population. In addition, the present study sample was obtained from eight different regions in China, including the coastal and inland regions and developed and underdeveloped regions, with a wide geographical span, making this an ideal representation of the Chinese population. Finally, the interaction between total sleep time and napping duration, total sleep time and blood pressure, and blood glucose and BMI were investigated, and a reference for individuals with diverse specific conditions was provided to control for the appropriate sleep duration.

The correlation between sleep and kidney function can be explained in the following ways: First, both short and long sleep durations can result in systemic inflammation, which may account mainly for the association with increased UACR levels. Long sleep durations are associated with subclinical inflammation and increased arterial stiffness [43–46], while sleep curtailment increases proinflammatory cytokine [47], high-sensitivity C-reactive protein [48], and white blood cell [49] levels, which reflects systemic inflammation, causes glomerular endothelial dysfunction, and consequently leads to albuminuria [50, 51]. Second, changes in the sympathetic nervous system influenced by higher or lower sleep durations may cause kidney function decline [52]. Sleep regulates the activity of the hypothalamic pituitary adrenal (HPA) axis. The activity of the HPA axis is reduced during sleep onset and the early stages of sleep, and it is activated during the latter stages of sleep, such as the rapid eye movement (REM) stage [46, 53, 54]. Therefore, extreme short sleep durations may weaken the inhibitory effect of early-stage sleep on the HPA axis, while extremely long sleep durations may enhance the activation effect of REM stage on the HPA axis, thereby keeping the activity of the HPA axis at a higher level, which has adverse effects on metabolic health. Third, sleep deprivation in humans reduces plasma renin, angiotensin and aldosterone levels, which is associated with the increased urinary excretion of sodium and potassium [55, 56]. In addition, the normal nocturnal reduction in blood pressure is attenuated. Related animal experiments have shown that sleep deprivation causes increased sympathetic nerve activity and reduced plasma angiotensin II levels [57]. Forth, regarding the association between napping and microalbuminuria, changes in circadian rhythms were speculated to contribute to the effect of sleep on kidney function. Animal models were constructed by mutating the circadian regulatory gene casein kinase-1ɛ, and a related experiment revealed that the animals that were heterozygous for the mutation exhibited phase-advanced and shortened circadian rhythms. These animals were shown to develop albuminuria, renal tubular atrophy and cardiac dysfunction [58]. This finding was supported by these present findings, in which daytime napping could not compensate for the lack of sleep at night when the total sleep duration was equal, and by the results reported by Sasaki *et al*. [26], in which short sleep duration was more likely to affect kidney health in shift workers. Fifth, different distributions of sleep-disordered breathing (SDB) [59, 60] and restless leg syndrome (RLS) [61, 62] in several sleep duration groups may also be a reason for the findings, according to previous reports.

The major strength of the present study was the large sample of the general population from eight areas of China, which made these present results representative and statistically significant and provided the possibility for the subsequent subgroup analysis of interactions. The REACTION study was an epidemiological investigation of tumors. In addition, detailed medical and drug-use histories were collected during the questionnaire process. In this manner, subjects with primary kidney diseases and those who used ACEI/ARB drugs could be excluded in the subsequent analyses to obtain more reliable results. However, the present study also has several limitations. First, sleep duration was determined according to a self-reported questionnaire, which is similar to many prior epidemiological studies, and this was not measured objectively. In addition, sleep quality could not be evaluated, and the prevalence of SDB or RLS could not be determined. Second, the cross-sectional study design prevented the investigators from establishing a causal relationship between sleep and kidney function. Third, data regarding protein intake on the previous day and the interval between the last meal and sleep were not available, which may affect the UACR level. Fourth, the UACR levels were determined by a single measurement, but the detection methods of the eight centers were different. Hence, several outcomes were set, and the UACR values were divided according to the quartile division in the center to which the subject belonged to in order to estimate the UACR level in the logistic regression.

In summary, the goal of the present study was to demonstrate the relationship between sleep duration, daytime napping duration, UACR and eGFR in a middle-aged and apparently healthy Chinese population. An explanation was provided for the present epidemiological investigation regarding the controversial inconsistent results of the relationship between sleep duration and eGFR levels. Further cohort studies should be conducted to confirm these conclusions.

## Supporting information

S1 Questionnaire English questionnaire of REACTION study. (DOCX)

S2 Questionnaire Chinese questionnaire of REACTION study. (DOCX)

S1 Dataset Raw data of present study. (SAV)
