# Supplementary material for: Self-reported sleep duration and daytime napping are associated with renal hyperfiltration and microalbuminuria in an apparently healthy Chinese population
Source: PLoS One. 2019 Aug 30;14(8):e0214776. doi: 10.1371/journal.pone.0214776 (PMC6716775; doi:10.1371/journal.pone.0214776)
Supplement: S1 Questionnaire — (DOCX) [file pone.0214776.s001.docx]

Risk Evaluation of cAncers in Chinese diabeTic Individuals: A lONgitudinal(REACTION) Study.

The questionnaire of baseline.

**Bar code paste place**

**Whether diabetes has been diagnosed: YES NO**

**DATE：**

**Name of the investigator:**

**PART 1 Basic Information**

**A. Subject Identification**

**1. Name：**

**2. Gender：1 = male， 2 = female**

**3. Birth date：** | | | | | **year** | | | **month** | | | **day**

**4. [Ancestral](C:/Users/tyyej/AppData/Local/youdao/dict/Application/7.5.1.0/resultui/dict/?keyword=ancestral)[home](C:/Users/tyyej/AppData/Local/youdao/dict/Application/7.5.1.0/resultui/dict/?keyword=home)：** **Birthplace：** **Living place：**

**5. Race：1.Han nationality 2. others （Please note** _**）**

**6. ID：**| | | | | | | | | | | | | | | | | | |

**7. TEL：**

**8. Mobile phone：**

**9. Address：**

**10. Family information：**

**Father： Name** **Birth date**| | | | |**year**| | |**month**| | |**day**

**Mother： Name** **Birth date**| | | | |**year**| | |**month**| | |**day**

**Spouse： Name** **Birth date**| | | | |**year**| | |**month**| | |**day**

**Children1：**

**Gender** **Name** **Birth date**| | | | |**year**| | |**month**| | |**day**

**Children2：**

**Gender** **Name** **Birth date**| | | | |**year**| | |**month**| | |**day**

**Children3：**

**Gender** **Name** **Birth date**| | | | |**year**| | |**month**| | |**day**

**Children4：**

**Gender** **Name** **Birth date**| | | | |**year**| | |**month**| | |**day**

**B. History of Diabetes**

**Have you ever been diagnosed with diabetes by a doctor?**

1.YES 2.NO

－If NO, turn to **C. History of Tumor.**

－If YES, please continue with the following questions.

Type of diabetes： 1. TYPE 1 2. TYPE 2 3. Gestational diabetes 4. Other types 5. Unknown

Date of first diagnosis：| | | | |year| | |month| | |day

Diagnose by OGTT( or Steamed Bread Meal Test)? 1.YES 2.NO

If YES, at that time, your fasting blood glucose is mmol/L，postprandial(2h) blood glucose is mmol/L，HbA1c is %

Current treatment measures (within 1 year)：

a. **Currently**, do you follow the doctor's advice on diet： 1.YES 2.NO

b. **Currently**, do you exercise properly as advised by your doctor： 1.YES 2.NO

c. **Currently**, do you take hypoglycemic drugs regularly： 1.YES (Fill in the name and dose in question 4 of part 2) 2.NO

d. **Currently**, do you use insulin： 1.YES (Fill in the name and dose in question 4 of part 2) 2.NO

e. **Currently**, do you take Chinese Traditional Medicine：1.YES (Fill in the name and dose in question 4 of part 2) 2.NO

**C History of Tumor**

**Do you ever had tumor before？**  1.YES 2.NO

－If NO, turn to **D. History of Surgery.**

－If YES, please continue with the following questions.

**Tumor site（If you have malignant tumor, please circle the letters corresponding to the following parts）:**

a. Liver b. Pancreas c. Stomach d. Intestine e. Breast

f. Cervical g. Endometrial h. Prostate gland i. Bladder j. Kidney k. Lung

l. Lymphoma m. Leukemia n. Others (Please note： )

|  | | **Site 1：** | **Site 2：** | **Site 3：** |
| --- | --- | --- | --- | --- |
| **Diagnosis date** | |  |  |  |
| **Diagnosed by which hospital** | |  |  |  |
| **Diagnostic evidence** | |  |  |  |
| **pathologic types** | |  |  |  |
| **pathological staging** | |  |  |  |
| **Whether it transfer** | |  |  |  |
| **Transfer to which organ** | |  |  |  |
| **Surgery** | **The operation name** |  |  |  |
|  | **Date of surgery** |  |  |  |
|  | **In which hospital** |  |  |  |
| **Radiation therapy** | **Treatment date** |  |  |  |
|  | **In which hospital** |  |  |  |
| **chemotherapy** | **Treatment date** |  |  |  |
|  | **In which hospital** |  |  |  |

1. **History of Surgery**

Have you had any other surgery? 1.YES 2.NO

－If NO, turn to **PART 2. Health Situation.**

－If YES, please continue with the following questions.

Surgical site（If you have received surgical treatment, please circle the letters corresponding to the following parts）：

a. Liver b. Pancreas c. Stomach d. Intestine e. Breast

f. Cervical g. Endometrial h. Prostate gland i. Bladder j. Kidney k. Lung

l. Lymphoma m. Leukemia n. Others (Please note： )

|  | **Site 1：** | **Site 2：** | **Site 3：** |
| --- | --- | --- | --- |
| **Operation name** |  |  |  |
| **Diagnosis date** |  |  |  |
| **Diagnosed by which hospital** |  |  |  |

**PART 2. Health Situation**

**QUESTION 1：Basic Information**

**Marital status:**

1. Married 2. Unmarried 3. Cohabitation 4. Widowed 5. Separation 6. Divorced 7.Others

**Who are you currently living with?**

1. Live with spouse and children 2. Live with spouse only 3. Live with children only
2. Live with others 5. Live alone: Since when? ______________________

**Current residence:**

1.Own home 2.Children's home 3.Home of other relatives 4.Nursing home 5.Others (Please note： )

**Current career:**

1.Worker 2.Peasant 3.Soldier 4.Cadre 5.Scientific, technical or medical personnel, teachers 6.Individual trader

7.Business and enterprise administration (service personnel) 8.housewife 9.Retirees 10.Unemployed personnel

11.Others (Please note： )

**Education level:**

1.Illiterate, semiliterate 2.primary school 3.Junior high school 4.High school or technical secondary school 5.Junior college or above

**QUESTION 2：Do you have any of the following clearly diagnosed diseases?**

Do you suffer from any of the following diseases?If yes, please provide the date (year/month) of each diagnosis and the name of the hospital where the diagnosis was made (if more than once, please list them one by one)

**1.Cardiovascular system：**

***a. Myocardial infarction：***

1.YES (Date of diagnosis________ Which hospital ) 2.NO

***b. Stroke：***

1.YES (Date of diagnosis________ Which hospital ) 2.NO

***c. Coronary heart disease：***

1.YES (Date of diagnosis________ Which hospital ) 2.NO

***d. Hypertension：***

1.YES (Date of diagnosis________ Which hospital ) 2.NO

***e. Arterial disease of lower extremity：***

1.YES (Date of diagnosis________ Which hospital ) 2.NO

***f. retinopathy：***

1.YES (Date of diagnosis________ Which hospital ) 2.NO

***g. Hyperlipidemia:***

1.YES (Date of diagnosis________ Which hospital ) 2.NO

**2.Digestive system：**

***2.1 Liver:***

***a. Fatty liver disease：***

1.YES (Date of diagnosis________ Which hospital ) 2.NO

***b. Viral hepatitis：***

1.YES (Date of diagnosis________ Which hospital

Type：Hepatitis B Hepatitis C Others Unknown ) 2.NO

***c. Hepatic cirrhosis：***

1.YES (Date of diagnosis________ Which hospital ) 2.NO

***d. Autoimmune liver disease: (e.g. Autoimmune hepatitis, primary biliary cirrhosis, primary sclerosing cholangitis, etc.)***

1.YES (Date of diagnosis________ Which hospital ) 2.NO

***e. Other liver diseases (e.g. benign liver tumors):***

1.YES (Date of diagnosis________ Which hospital ) 2.NO

***2.2 Biliary:***

***a. Cholecystitis：***

1.YES (Date of diagnosis________ Which hospital Surgical treatment or not：YES NO ) 2.NO

***b. Gallstones：***

1.YES (Date of diagnosis________ Which hospital Surgical treatment or not：YES NO ) 2.NO

***c. Gallbladder polyps：***

1.YES (Date of diagnosis________ Which hospital Surgical treatment or not：YES NO ) 2.NO

***d. Other biliary diseases：***

1.YES (Date of diagnosis________ Which hospital ) 2.NO

***2.3 pancreas:***

***a. Acute pancreatitis：***

1.YES (Date of diagnosis________ Which hospital Surgical treatment or not：YES NO ) 2.NO

***b. Chronic pancreatitis：***

1.YES (Date of diagnosis________ Which hospital Surgical treatment or not：YES NO ) 2.NO

***c. Other pancreas diseases：***

1.YES (Date of diagnosis________ Which hospital ) 2.NO

***2.4 Gastrointestinal diseases:***

***a. Chronic gastroenteritis：***

1.YES (Date of diagnosis________ Which hospital ) 2.NO

***b. Gastroduodenal ulcer：***

1.YES (Date of diagnosis________ Which hospital Surgical treatment or not：YES NO ) 2.NO

***c. Other gastrointestinal diseases：***

1.YES (Date of diagnosis________ Which hospital ) 2.NO

**3.Kidney disease：**

***a. Kidney stones：***

1.YES (Date of diagnosis________ Which hospital ) 2.NO

***b. Renal cyst：***

1.YES (Date of diagnosis________ Which hospital ) 2.NO

***c. Chronic nephritis：***

1.YES (Date of diagnosis________ Which hospital ) 2.NO

***d. Nephrotic syndrome：***

1.YES (Date of diagnosis________ Which hospital ) 2.NO

***e. Other kidney diseases：***

1.YES (Date of diagnosis________ Which hospital ) 2.NO

**4.Respiratory system：**

***a. Chronic bronchitis：***

1.YES (Date of diagnosis________ Which hospital ) 2.NO

***b. emphysema：***

1.YES (Date of diagnosis________ Which hospital ) 2.NO

***c. Other respiratory diseases：***

1.YES (Date of diagnosis________ Which hospital ) 2.NO

***d. Whether or not you have snored during night sleep over the past 12 months?***

1. Often 2. Occasionally 3. Never 4. Unknown

**5.Fracture**

1. YES

Site 1____________ Age 1____________ Reason 1_____________

Site 2____________ Age 2____________ Reason 2_____________

Site 3____________ Age 3____________ Reason 3_____________

2. NO

6**.Have you ever had schistosomiasis?**

－If NO, turn to **7. Other diseases.**

－If YES, please continue with the following questions.

Please indicate age of infection with schistosomiasis __________

Please indicate which of the following treatments you have received:

1. Anti-schistosomiasis drugs 2. Splenectomy treatment 3. Unknown

Please indicate whether you have ever been diagnosed with schistosomiasis liver disease: 1. YES 2. NO 3. Unknown

**7. Other diseases.(Except for diabetes)**

______________ (Date of diagnosis________ Which hospital )

_______________(Date of diagnosis________ Which hospital )

_______________(Date of diagnosis________ Which hospital )

**QUESTION 3：Family medical history - diabetes and cancer**

**Have any of your blood relatives (including those who have passed away) had malignant tumors or diabetes:**

1. YES 2.NO

－If NO, turn to **QUESTION 4.**

－If YES, please continue with the following questions.

(Multiple choices are available. If there is any disease, select "1" in the circle of "whether there is disease";If the disease does not exist, circle "2". If the affected persons are sons, daughters, brothers and sisters, please specify the number of affected persons in each option.If the patient is a parent, grandparent or grandparent, the number of patients should not be filled in.)

|  | Family medical history of tumor | | | | | | Family medical history of diabetes | | |
| --- | --- | --- | --- | --- | --- | --- | --- | --- | --- |
|  | Whether there is disease | | The number of patients | Site 1 | Site 2 | Site 3 | Whether there is disease | | The number of patients |
| Father | 1.YES | 2.NO |  |  |  |  | 1.YES | 2.NO |  |
| Mother | 1.YES | 2.NO |  |  |  |  | 1.YES | 2.NO |  |
| Son | 1.YES | 2.NO |  |  |  |  | 1.YES | 2.NO |  |
| Daughter | 1.YES | 2.NO |  |  |  |  | 1.YES | 2.NO |  |
| Brother | 1.YES | 2.NO |  |  |  |  | 1.YES | 2.NO |  |
| Sister | 1.YES | 2.NO |  |  |  |  | 1.YES | 2.NO |  |
| Grandfather(Father's father) | 1.YES | 2.NO |  |  |  |  | 1.YES | 2.NO |  |
| Grandmother(Father's mother) | 1.YES | 2.NO |  |  |  |  | 1.YES | 2.NO |  |
| Grandfather(mother's father) | 1.YES | 2.NO |  |  |  |  | 1.YES | 2.NO |  |
| Grandmother(mother's mother) | 1.YES | 2.NO |  |  |  |  | 1.YES | 2.NO |  |

**QUESTION 4.**

**Have you ever received hypoglycemic therapy since your diagnosis of diabetes?**

1. YES 2.NO

－If NO, turn to **QUESTION 5.**

－If YES, please indicate which type: 1. Oral drugs 2. Insulin 3. Oral drugs + insulin

**Have you taken any hypoglycemic drugs or insulin injections in a year?**

－If NO, turn to **QUESTION 5.**

－If YES, please describe in detail the name of the medication you are taking, the daily dose, the date you started taking it, and the cumulative duration of the medication.

|  | Drug category | Drug name | Drug dose (/ day) | Start date (year/month) | Duration (month) |
| --- | --- | --- | --- | --- | --- |
| 1 | Sulfonylureas |  |  |  |  |
| 2 | Glinides |  |  |  |  |
| 3 | Biguanides |  |  |  |  |
| 4 | Glycosidase inhibitors |  |  |  |  |
| 5 | Thiazolidinedione |  |  |  |  |
| 6 | Regular Insulin |  |  |  |  |
| 7 | Insulin glargine |  |  |  |  |
| 8 | GLP1 analogues |  |  |  |  |
| 9 | DPP4 inhibitor |  |  |  |  |
| 10 | Chinese traditional medicine |  |  |  |  |
| 11 | Others |  |  |  |  |

| **Table 1 General list of diabetes drugs** | |
| --- | --- |
| Sulfonylureas | - Glyburide - Gliclazide - Glipizide - Gliquidone - Glimepiride - Tolbutamide |
| Glinides | - Repaglinide - Nateglinide |
| Biguanides | - Melbine - Phenformin |
| Glycosidase inhibitors | - Acarbose - Voglibose |
| Thiazolidinedione | - Rosiglitazone - Pioglitazone |
| Regular Insulin | - Insulin aspart - Insulin lispro - Human insulin - Animal insulin |
| Insulin glargine | - Insulin glargine |
| GLP1 analogues | - Liraglutide |
| DPP4 inhibitor | - Vildagliptin - Januvia |
| Chinese traditional medicine | - Xiaoke pill - Jinqi hypoglycemic tablet |
| Others |  |

PS: Ventamine is a compound of metformin and rosiglitazone.Insulin pump users were recorded as normal insulin group

**QUESTION 5. Have you taken any other medication in the last two weeks (including today)?(except hypoglycemic drugs)**

－If NO, turn to **QUESTION 6.**

－If YES, please describe in detail the name of the medication you are taking, the daily dose, the date you started taking it, and the cumulative duration of the medication

|  | Drug category | Drug name | Drug dose (/ day) | Start date (year/month) | Duration (month) |
| --- | --- | --- | --- | --- | --- |
| 1 |  |  |  |  |  |
| 2 |  |  |  |  |  |
| 3 |  |  |  |  |  |
| 4 |  |  |  |  |  |
| 5 |  |  |  |  |  |

**QUESTION 6. Smoking situation. (definition of daily smoking: 1 cigarette per day or 7 cigarettes per week for at least 6 months)**

**6.1 Do you smoke daily now?**

1. NO

2. Occasionally (not every day)

3. YES, every day or almost every day

**6.2 How about your smoking habits in the past?**

1. Never smoke (Turn to **QUESTION 6.8**)

2. Used to smoke occasionally (less than once a day) (**Turn to QUESTION 6.8**)

3. Used to smoke daily

**6.3 How many years since you quit smoking?** ____________years.

**6.4 How much you smoke now or before you quit?**

Cigarettes _______________/day

Cigar /day

**Hand-rolled cigarette**  **kg/month**

**6.5 Do you inhale smoke when you smoke?**   **1.YES 2.NO**

**6.6 How old were you when you started smoking daily？**  **years old**

**6.7 How many years have you been in the habit of smoking daily?**  ________**years**

**Passive smoking:**

**6.8 Did anyone smoke in your family before you were 18?**   **1.YES 2.NO**

**6.9 Does anyone in your family smoke now?**

**1.YES**

**You currently have a total of ________ family member who smokes.**

**Your family smokes a total of ________ cigarettes a day around you.**

**This situation last for_________year(s).**

**On average, you're exposed to this smoke situation_________ days a week.**

**You're exposed to this smoke situation_________ minutes a day.**

**2. NO**

**6.10 Have colleagues smoked in front of you (to make you feel the smoke inhalation)？**

**1.YES**

**A total of _________ people smoked in the workplace**

**The total number of cigarettes smoked by your coworkers in front of you each day is about _______**

**This situation last for_________year(s).**

**On average, you're exposed to this smoke situation_________ days a week.**

**You're exposed to this smoke situation_________ minutes a day.**

**2. NO**

**QUESTION 7. Drinking Habits (Ask about your consumption of beer, white wine, yellow wine, wine, and other alcoholic beverages in the past 12 months.Regular drinking was defined as drinking at least once a week for at least six months)**

**7.1 Do you drink regularly now?**

1. NO

2. Occasionally (not every week)

3. YES, every week or almost every week(Turn to **QUESTION 7.4**)

**7.2 How about your drinking habits in the past?**

1. Never drink (Turn to **QUESTION 8**)

2. Used to drink occasionally (less than once a week) (Turn to **QUESTION 8**)

3. Used to drink every week

**7.3 How many years since you quit drinking?** ____________years.

**7.4 How often and how much do you drink now or before you quit?**

White wine (whisky)： kg/each time ； times/week Yellow rice wine (rice wine) ： kg/each time ； times/week

Red Wine： kg/each time ； times/week

Beer (750ml/bottle)： bottles/each time ； times/week

Other types ： kg/each time ； times/week

**7.5 How long have you been drinking regularly?**  Years

**7.6 Have you had any heavy drinking or drunkenness in the past year (as follows)：**

A. Drink only white wine, 0.15kg or more at one time

1. YES _______kg/one time

2. NO

B. Drink only yellow rice wine, 0.25kg or more at one time

1. YES _______kg/one time

2. NO

C. Drink only red wine, 0.25kg or more at one time

1. YES _______kg/one time

2. NO

D. Drink only Beers (750ml/bottle), 2 bottles or more at one time

1. YES _______bottle(s)/one time

2. NO

E. Drink two or more types of alcohol at a time

1. YES _______kg/one time white wine

_______kg/one time yellow rice wine

_______kg/one time red wine

_______bottle(s)/one time beers

2. NO

**7.7 The number of times you have been drinking heavily or getting drunk in the past year：**__________/month.

**QUESTION 8. Tea Drinking Habits(Excluding tea beverage)**

**8.1 Which of the following best reflects your tea drinking in the past year？** 1. Never or almost never (Turn to **QUESTION 9**)

2. Occasional drinks (i.e. 1-3 drinks a month at most, less than once a week) (Turn to **QUESTION 9**)

3. I used to drink tea, but now I don't

4. Drink tea regularly now (at least one day per week) (Turn to **QUESTION 8.3**) **8.2 You have stopped drinking tea for _______ years. ( fill “0” for less than one year)**

**8.3 What is the amount and frequency of you drinking tea now or before you stop drinking tea?(according to the 150ml cup, please choose the kind of tea you drink most often.)**

Green tea/jasmine tea/longjing/white tea ________cup(s)/day ______day(s)/week

Oolong tea (tieguanyin, narcissus, etc.) ________cup(s)/day ______day(s)/week

Black tea (including brick tea, pu 'er tea) ________cup(s)/day ______day(s)/week

Others( Please note_____________) ________cup(s)/day ______day(s)/week

**8.4 in the past year, what is your average monthly tea consumption?**

_________kg/month

**8.5 at what age do you habitually drink tea (at least one day a week)?** _________years old

**QUESTION 9. Occupational and environmental risk factors**

| 9.1 | Have you been involved in the paint industry in the past year? | 1. Often 2. Occasionally 3. Never 4. Unknown |
| --- | --- | --- |
| 9.2 | Have you been exposed to radiation in the past year? | 1. Often 2. Occasionally 3. Never 4. Unknown |
| 9.3 | Have you been exposed to any chemicals in the past year? | 1. Often 2. Occasionally 3. Never 4. Unknown |
| 9.4 | Have you decorated your home or workplace in the past year? | 1.YES 2.NO 3.Unknown |
| 9.5 | Have you lived near any chemical or nuclear plants in the past year? | 1.YES 2.NO 3.Unknown |

**QUESTION 10. The Weight Change**

| 10.1 | When was the last time you weighed yourself before today? | | | | | 1. Never measured  2. Within 1 month  3. Within 6 months  4. 6 to 12 months  5. 12 months ago  6. Don't remember | | | | |
| --- | --- | --- | --- | --- | --- | --- | --- | --- | --- | --- |
| 10.2 | Compared to the last year, your weight is | | | | | 1. Reduce  2. Add  3. Basically unchanged  4. Not clear | | | | |
| 10.3 | Have you taken any steps to change your weight in the past 12 months? | | | | | 1. Try to loss weight  2. Try to gain weight  3. Not trying to change | | | | |
| 10.4 | If you are trying to lose weight, what methods do you use? (multiple choices) | | | | | 1. Control your diet  2. Exercise  3. The drugs  4. Others (please note) | | | | |
| 10.5 | Please recall the different stages of weight below | | | | | For females only | | | | |
| Age(years) | 0 | 20 | 30 | 40 | 50 | 60 | 70 | >70 | before fertility | after fertility |
| （Kg） |  |  |  |  | |  |  |  |  |  |

**QUESTION 11. Emotional situation**

| 11．1 | In the last 2 weeks, have you had no interest in anything? | 1 no  2 a few days, but not many  3 more than a week  4 almost every day  5 Unclear  6 refuse to answer |
| --- | --- | --- |
| 11．2 | Have you been feeling down, depress or hopeless in the last 2 weeks? | 1 no  2 a few days, but not many  3 more than a week  4 almost every day  5 Unclear  6 refuse to answer |
| 11．3 | Have you had trouble sleeping, failed to fall asleep, or sleep too much in the last 2 weeks? | 1 no  2 a few days, but not many  3 more than a week  4 almost every day  5 Unclear  6 refuse to answer |
| 11．4 | In the last 2 weeks, have you felt tired and weak? | 1 no  2 a few days, but not many  3 more than a week  4 almost every day  5 Unclear  6 refuse to answer |
| 11．5 | Have you lost your appetite or eaten too much in the last 2 weeks? | 1 no  2 a few days, but not many  3 more than a week  4 almost every day  5 Unclear  6 refuse to answer |
| 11．6 | In the last 2 weeks, have you been dissatisfied with yourself, or felt like a loser, or felt disappointed your family? | 1 no  2 a few days, but not many  3 more than a week  4 almost every day  5 Unclear  6 refuse to answer |
| 11．7 | In the last 2 weeks, have you felt unable to concentrate on something, like reading a book, reading a newspaper or watching TV? | 1 no  2 a few days, but not many  3 more than a week  4 almost every day  5 Unclear  6 refuse to answer |
| 11．8 | In the last 2 weeks, have you felt yourself moving or speaking slowly enough to get noticed, or, conversely, fidgeting, fidgeting, and moving around more easily than usual? | 1 no  2 a few days, but not many  3 more than a week  4 almost every day  5 Unclear  6 refuse to answer |
| 11．9 | In the last 2 weeks, have you had any thoughts of suicide or hurting yourself? | 1 no  2 a few days, but not many  3 more than a week  4 almost every day  5 Unclear  6 refuse to answer |
| 11.10 | If you have the above problems (11.1-11.9), do they have any impact on your daily work, family life and getting along with others? | 1 has no effect  2 some influence  3 Three makes a big difference  4 great impact  5 Unclear |
| 11．11 | How was your relationship with your family (parents, spouse, children) over the past 12 months? | 1 Excellent  2 good  3 general  4 poor  5 very poor  6 living alone without family  7 refuse to answer |
| 11．12 | In the past 12 months, have you had any major setbacks? | 1 YES  2 NO  3 refuse to answer |
| 11．13 | In general, have you been satisfied with your life over the past 12 months? | 1 very satisfied  2 happy  3 general  4 unsatisfied  5 very unsatisfied  6 refuse to answer |

**QUESTION 12. Diet Situation**

| 12.1 | How many meals a day have you normally eaten in the past 12 months? | | | | /day | | | | | |
| --- | --- | --- | --- | --- | --- | --- | --- | --- | --- | --- |
|  | How many days in a week have you normally eaten breakfast in the past 12 months? | | | | day(s) | | | | | |
|  | How many days in a week have you normally eaten lunch in the past 12 months? | | | | day(s) | | | | | |
|  | How many days in a week have you normally eaten supper in the past 12 months? | | | | day(s) | | | | | |
|  | How many days in a week have you normally eaten [midnight](C:/Users/tyyej/AppData/Local/youdao/dict/Application/7.5.1.0/resultui/dict/?keyword=midnight)[snack](C:/Users/tyyej/AppData/Local/youdao/dict/Application/7.5.1.0/resultui/dict/?keyword=snack) in the past 12 months? | | | | day(s) | | | | | |
|  | | | | | Dining place | | | | | |
|  |  |  |  |  | a.  Home | | b. [Canteen](C:/Users/tyyej/AppData/Local/youdao/dict/Application/7.5.1.0/resultui/dict/?keyword=canteen) | | C. restaurant | |
| 12.2 | How many days a week have you normally eaten breakfast at different eating places in the past 12 months? | | | | __day(s) | | __day(s) | | __day(s) | |
| 12.3 | How many days a week have you normally eaten lunch at different eating places in the past 12 months? | | | | __day(s) | | __day(s) | | __day(s) | |
| 12.4 | How many days a week have you normally eaten supper at different eating places in the past 12 months? | | | | __day(s) | | __day(s) | | __day(s) | |
| Please recall if you have eaten any of the following foods in the past 12 months and estimate the frequency and amount of each. | | | | | | | | | | |
|  | |  | a. Eaten or not | b. How many times a day/week/month/year do you eat this food? | | | | | | c.amount |
|  | |  | 1 YES  2 NO | b 1  times/day | b 2  times/week | b 3  times/month | | b4  times/year | | kg |
| 12.5 | | Rice, flour, coarse grains and other cereals (recorded by raw weight) |  |  |  |  | |  | | kg |
| 12.6 | | Potato (potato/taro/sweet potato) |  |  |  |  | |  | | kg |
| 12.7 | | Pork (recorded by raw weight) |  |  |  |  | |  | | kg |
| 12.8 | | Meat of cattle, sheep, etc  (recorded by raw weight) |  |  |  |  | |  | | kg |
| 12.9 | | Poultry such as chickens, ducks and geese |  |  |  |  | |  | | kg |
| 12.10 | | Aquatic products  (fish and shrimp by raw weight) |  |  |  |  | |  | | kg |
| 12.11 | | Fresh vegetables |  |  |  |  | |  | | kg |
| 12.12 | | Fresh fruit |  |  |  |  | |  | | kg |
| 12.13 | | Fresh vegetable juice (250ml/ cup) |  |  |  |  | |  | | cup(s) |
| 12.14 | | Eggs |  |  |  |  | |  | |  |
| 12.15 | | Dairy products (fresh milk) |  |  |  |  | |  | | kg |
| 12.16 | | Soybean products ( tofu) |  |  |  |  | |  | | kg |
| 12.17 | | Fried food (deep-fried dough sticks, oil cakes, etc.) |  |  |  |  | |  | | kg |
| 12.18 | | Fruit and vegetable juice/fruity drink (250ml/ cup) |  |  |  |  | |  | | cup(s) |
| 12.19 | | Carbonated drinks (250ml/ cup) |  |  |  |  | |  | | cup(s) |
| 12.20 | | pastry |  |  |  |  | |  | | kg |
| 12.21 | | pickles |  |  |  |  | |  | | No need to fill |
| 12.22 | | kimchi |  |  |  |  | |  | |  |
| 12.23 | | Fermented bean curd |  |  |  |  | |  | |  |
| 12.24 | | coffee |  |  |  |  | |  | |  |
| 12.25 | | animal's gut |  |  |  |  | |  | |  |
| 12.26 | | Nutrient supplements (e.g. vitamins, minerals, etc.) |  |  |  |  | |  | |  |

Table 2. Food conversion

Staple food conversion

| Food | unit | The equivalent of raw rice or pasta in grams | kg |
| --- | --- | --- | --- |
| Rice | 1 small standard bowl (diameter 12cm) | 75 | 0.075 |
|  | 1 big standard bowl (diameter 16cm) | 150 | 0.15 |
| Rice porridge | 1 small standard bowl (diameter 12cm) | 30 | 0.03 |
|  | 1 big standard bowl (diameter 16cm) | 50 | 0.05 |
| Steamed bread | 1 (need to be converted according to size) | 100 | 0.1 |

Dairy products conversion

| Dairy products | How much this product equal to 1kg of fresh milk |
| --- | --- |
| Fresh cow milk (goat's milk) | 1kg |
| yogurt | 1kg |
| Milk powder | 0.15kg (About 1 1/2 TSP) |
| cheese | 0.1kg |

Soybean products conversion

| Soybean products | How much this product equal to 1kg of BeiDouFu |
| --- | --- |
| BeiDouFu | 1kg |
| Lactone tofu | 2.4kg |
| Bean curd cake | 0.75kg |
| Shredded Dried Tofu | 0.5kg |
| Bean curd stick | 0.25kg |
| Soya-bean milk | 5kg |

**QUESTION 13. physical activity and sleep in the past seven days**

**13.1 Do you have a job? (full-time or part-time)**

1.YES 2.NO

－If YES, please your profession ________________；

When you at work：

1.Barely exercise (office, etc.) You do this _____day(s) a week, _____hour(s) a day.

2.Mild exercise (assembly line work, etc.) You do this _____day(s) a week, _____hour(s) a day.

3.Moderate exercise (installers, porters, etc.) You do this _____day(s) a week, _____hour(s) a day.

4.Heavy exercise (steelmaking, agriculture, casting, etc.) You do this _____day(s) a week, _____hour(s) a day.

**13.2 Please provide information about strenuous physical activity in your spare time outside of work during the past seven days. (strenuous activity refers to many activities that require you to have difficulty breathing, such as lifting weights, playing basketball, swimming, running, etc.Please only count each activity that lasts for 10 minutes or more.)**

1.YES You do this _____day(s) a week, _____hour(s) a day.

2. NO

**13.3 Please provide information about moderate physical activity in your spare time outside work in the past seven days. (moderate physical activity requires you to breathe a little more [laboriously](C:/Users/tyyej/AppData/Local/youdao/dict/Application/7.5.1.0/resultui/dict/?keyword=laboriously&lang=en) than normal activities, such as jogging, table tennis, tai chi, etc., but does not include walking.Please only count each activity that lasts for 10 minutes or more.)**

1.YES You do this _____day(s) a week, _____hour(s) a day.

2. NO

**13.4 please provide information about your walking activities in the past seven days (including any form of walking at work and in your spare time).Please only count each walk lasting 10 minutes or more.)**

1.YES You do this _____day(s) a week, _____hour(s) a day. 2. NO

**13.5 please recall whether you have carried out the following activities in the past 12 months and estimate the frequency and duration of each activity.**

|  | a. I've done this in the last 12 months | b. How many times a day/week/month/year did you carry out this activity? | | | | How long did the activity last? |
| --- | --- | --- | --- | --- | --- | --- |
|  | 1 YES  2 NO | b 1  times/day | b 2  times/week | b 3  times/month | b4  times/year |  |
| Yoga |  |  |  |  |  | \| \| \|h\| \| \|min |
| Tai chi |  |  |  |  |  | \| \| \|h\| \| \|min |
| Aerobics |  |  |  |  |  | \| \| \|h\| \| \|min |
| Dance |  |  |  |  |  | \| \| \|h\| \| \|min |
| Fitness |  |  |  |  |  | \| \| \|h\| \| \|min |
| Others_______ |  |  |  |  |  | \| \| \|h\| \| \|min |
| Others_______ |  |  |  |  |  | \| \| \|h\| \| \|min |

**13.6 please provide your sitting situation in the past week (including sitting at work and leisure)**

Monday to Friday：

1.YES You do this _____day(s) a week, _____hour(s) a day. 2.NO

Saturday and Sunday：

1.YES You do this _____day(s) a week, _____hour(s) a day. 2.NO

**13.7 your daily sleep duration during the past 7 days**

1. I sleep well, I usually take a daytime nap for____hour(s) and ____minutes, I sleep from_______to_______ at night

2. I don't sleep well, I usually take a daytime nap for____hour(s) and ____minutes, I sleep from_______to_______ at night

3. I need sleeping pills to help me sleep, I usually take a daytime nap for____hour(s) and ____minutes, I sleep from_______to_______ at night

**13.8 what was the average amount of television time you watched per day over the past 12 months?** ______hour(s)________minutes(s)

**13.9 what is the average amount of time you have used your computer per day in the past 12 months?** ______hour(s)________minutes(s)

**13.10 what is the average amount of time you have spent reading (paper books) per day in the past 12 months?** ______hour(s)________minutes(s)

**13.11 What is the average amount of time you have spent playing video games (excluding computer games) per day in the past 12 months?** ______hour(s)________minutes(s)

**13.12 Do you usually use a mobile phone?**

1. YES, How many years have you used your mobile phone?________years

2. Occasionally

3. Never

**QUESTION 14. Please provide information about your fertility.**

**14.1 You are male.**

**How many children have you had?______ . Among them, ____are(is) boy(s),____are(is) girl(s)**

**Did you have any giant fetuses (birth weight > 4kg) before?**

**1.YES. How many giant fetuses have you had?______ . Among them, ____are(is) boy(s),____are(is) girl(s)**

**2. NO**

14.2 You are female.

● How many times have you been pregnant?______

● how many abortions have you had?________, including spontaneous abortion______time(s), induced abortion ________time(s).

● How many children have you had?_______. Among them, ____are(is) boy(s),____are(is) girl(s)

● Did you have any giant fetuses (birth weight > 4kg) before?

1.YES. How many giant fetuses have you had?______ . Among them, ____are(is) boy(s),____are(is) girl(s)

2. NO

● have you ever had gestational hypertension during pregnancy: 1. YES 2. NO

● have you ever had gestational hyperglycemia during pregnancy: 1. YES 2. NO

● have you ever had ectopic pregnancy during your pregnancy:

1. YES. How many times________ 2. No

● Did you breastfeed your children?

1. YES Breastfeeding duration: on average______months per child.

2. NO

**Question 15 :(for women only) please provide information about your period.**

15.1 When was your first period ______(year)______(month)( you were_____years old)

15.2 are you still menstruating?

1. Yes, when was your last period_________（Turn to 15.5）

2.NO

15.3 please state when you are fully menopausal ______(year)______(month)( you were_____years old)

15.4 Did you have natural menopause?(if it is natural menopause, answer yes to this question, although you have subsequently had surgery involving your reproductive organs.)

1.YES

2.NO. Please describe the operation you had, the date (year/month), the reason and the name of the hospital where the operation was performed.

| operation | date (year/month) | reason | hospital |
| --- | --- | --- | --- |
|  |  |  |  |
|  |  |  |  |

15.5 have you ever taken birth control pills or used sex hormone replacement therapy?

1. YES. Please describe the name, daily dose, start time and cumulative duration of the medication.

| Medicine name | daily dose | start time | cumulative duration |
| --- | --- | --- | --- |
|  |  |  |  |
|  |  |  |  |

2. NO

**PART 3 Clinical examination**

**1．Blood pressure measurement (omron) Name of investigator**：

Have you taken any blood pressure medicine today? 1. YES 2. NO

Did you sit still for 5 minutes just now?

1. YES 2. NO(please sit quietly for 5 minutes and start measuring again)

Measuring time __ __：__ __ Hours/minutes）

Measured upper arm： 1. right 2. left

SBP1 /DBP1 mmHg HR1 /min

**（wait for 1 min）**

SBP2 /DBP2 mmHg HR2 /min

**（wait for 1 min）**

SBP3 /DBP3 mmHg HR3 /min

**2．Clinical measurement**

Height (accurate to 0.1cm) . cm

Weight (accurate to 0.1kg) . kg

Waist circumference (accurate to 0.1cm) . cm

Hip circumference (accurate to 0.1cm) . cm

**3. Electrocardiogram**  1. Done 2. Not done Name of investigator：

**4. Laboratory tests**

Blood sample collection:

Fasting or not: 1 YES 2 NO

Last meal date(month/day)____________________

Last meal time(hour/min)______________________

0min blood collection 1. Done 2. Not done

2h blood collection 1. Done 2. Not done

Do you take hypoglycemic drugs or use insulin today? 1 YES 2 NO

When did you take medicine(use insulin) last time： （hour/min）

**PART 4 Auxiliary examination**

**1. Cardiovascular and cerebrovascular complications of diabetes**

**1.1. Ultrasonic examination -- measurement of carotid internal media thickness (IMT) :**

Medial thickness of the left carotid artery mm；

Medial thickness of the right carotid artery mm；

Whether there is plaque： 1. YES

Site of the plaque

a.Left

1)homogeneity number

2)heterogeneity number

b.right

1)homogeneity number

2)heterogeneity number

2. NO

Name of investigator：

**1.2 vascular function examination (PWV, ABI, TBI)：**

1. Done 2.Not done Name of investigator：

**2. Diabetic retinopathy**

**Ophthalmoscope：** 1. Done 2.Not done Name of investigator：

**3. Diabetic nephropathy**

**UACR:** 1. Collected 2. Not collected Name of investigator：________

**4、Osteoporosis**

**Bone mineral density examination:**

1. Done 2.Not done Name of investigator：

Please circle the letter before the following results:

A.Normal bone density

B.Bone density loss

C.osteoporosis
