# Supplementary material for: Self-reported sleep duration and daytime napping are associated with renal hyperfiltration and microalbuminuria in an apparently healthy Chinese population
Source: PLoS One. 2019 Aug 30;14(8):e0214776. doi: 10.1371/journal.pone.0214776 (PMC6716775; doi:10.1371/journal.pone.0214776)
Supplement: S2 Questionnaire — (DOCX) [file pone.0214776.s002.docx]

中国2型糖尿病患者并发症发生风险的流行病学研究

基线问卷

**是否已诊断糖尿病: 是 否**

条形码黏贴处

**检查日期： 年 月 日**

**问卷医生姓名:**

**第一部分 基本信息**

**A 确认受检者身份**

1、姓名：

2、性别：1 = 男， 2 = 女

3、出生日期： | | | | | 年 | | | 月 | | | 日

4、祖籍： 出生地： 最长居住地：

5、民族：1.汉族 2. 其他 （请注明 _）

6、身份证号码：| | | | | | | | | | | | | | | | | | |

7、家庭电话：

8、个人手机：

9、详细家庭地址：

10、家庭信息：

父亲： 姓名 出生日期| | | | |年| | |月| | |日

母亲： 姓名 出生日期| | | | |年| | |月| | |日

配偶： 姓名 出生日期| | | | |年| | |月| | |日

子女： 性别 姓名 出生日期| | | | |年| | |月| | |日

性别 姓名 出生日期| | | | |年| | |月| | |日

性别 姓名 出生日期| | | | |年| | |月| | |日

性别 姓名 出生日期| | | | |年| | |月| | |日

**B 糖尿病病史**

**您是否被医生诊断过糖尿病：**

1.是 2.否

－如果否，请进入**C 肿瘤病史**。

－如果是，请继续以下问题。

请注明类型： 1. 1型 2. 2型 3. 妊娠糖尿病 4. 其他类型糖尿病 5. 不清楚

第一次诊断时间：| | | | |年| | |月| | |日

是否通过口服葡萄糖耐量试验（OGTT或馒头餐）确诊？ 1.是 2.否

如果是，当时空腹血糖 mmol/L，服糖后2小时血糖 mmol/L，糖化血红蛋白 %

**目前治疗措施（一年内）：**

a. **目前**是否按照医生建议控制饮食： 1.是 2.否

b. **目前**是否按照医生建议合理运动： 1.是 2.否

c. **目前**是否规律服降糖药： 1.是（将具体药名及剂量填入第二部分问题4） 2.否

d. **目前**是否规律使用胰岛素： 1.是（将具体药名及剂量填入第二部分问题4） 2.否

e. **目前**是否规律服用中药： 1.是（将具体药名及剂量填入第二部分问题4） 2.否

**C 肿瘤病史**

**您是否有过肿瘤病史？** 1.是 2.否

－如果否，请进入**D 手术史**。

－如果是，请继续以下问题。

**肿瘤部位（如患有恶性肿瘤，请在以下相应部位对应的字母上画圈，可多选）:**

a. 肝脏 b. 胰腺 c. 胃 d. 结直肠 e. 乳腺 f. 宫颈

g. 子宫内膜 h. 前列腺 i. 膀胱 j. 肾脏 k. 肺

l. 淋巴瘤 m. 白血病 n．其他(请注明： )

|  | | **部位一：** | **部位二：** | **部位三：** |
| --- | --- | --- | --- | --- |
| **确诊日期** | |  |  |  |
| **确诊医院** | |  |  |  |
| **确诊依据** | |  |  |  |
| **病理类型** | |  |  |  |
| **病理分期** | |  |  |  |
| **是否转移** | |  |  |  |
| **转移部位** | |  |  |  |
| **手术治疗** | **手术名称** |  |  |  |
|  | **手术日期** |  |  |  |
|  | **手术医院** |  |  |  |
| **放疗** | **治疗时间** |  |  |  |
|  | **治疗单位** |  |  |  |
| **化疗** | **治疗时间** |  |  |  |
|  | **治疗单位** |  |  |  |

**D 手术史**

**您是否接受过其他手术治疗？** 1.是 2.否

－如果否，请进入**第二部分：健康状况调查**。

－如果是，请继续以下问题。

**手术部位（如接受过手术治疗，请在以下相应部位对应的字母上画圈，可多选）:**

a. 肝脏 b. 胰腺 c. 胃 d. 结直肠 e. 乳腺 f. 宫颈

g. 子宫 h. 前列腺 i. 膀胱 j. 肾脏 k. 肺

n．其他(请注明： )

|  | **部位一：** | **部位二：** | **部位三：** |
| --- | --- | --- | --- |
| **手术名称** |  |  |  |
| **手术日期** |  |  |  |
| **手术医院** |  |  |  |

**第二部分 健康状况**

**问题1：基本情况**

**婚姻状况：**

1.已婚 2.未婚 3.同居 4.丧偶 5.分居 6.离异 7.其他

**目前居住情况：**

1.与配偶和子女住在一起 2.只与配偶住在一起 3.只与子女住在一起

4.同其他人居住 5.独自一人居住: 从 年 月开始独自居住

**目前居住地：**

1.自己的家 2.子女家 3.其他亲属家 4.养老院 5.其他（请注明： ）

**现在职业：**

1.工人 2.农民 3.军人 4.干部 5.科技人员、医务、教师 6.个体经商户

7.商业、企政（服务人员） 8.家庭妇女 9.离、退休人员 10.待业人员

11.其他(请注明： )

**文化程度：**

1.文盲、半文盲 2.小学 3.初中 4.高中或中专 5.大专及大专以上

**问题2：您是否患有明确诊断的以下疾病？**

请问您是否患有以下疾病；如果有，请提供每一疾病明确诊断的日期（年/月），以及得到明确诊断的医院名称（如果超过一次，请逐次列出）

**1.心血管系统：**

***a. 心肌梗死：*** 1.是 (诊断日期________ 医院名称 ) 2.否

***b. 脑卒中：*** 1.是 (诊断日期________ 医院名称 ) 2.否

***c. 冠心病：*** 1.是 (诊断日期________ 医院名称 ) 2.否

***d. 高血压：***  1.是 (诊断日期________ 医院名称 ) 2.否

***e. 下肢动脉病变：*** 1.是 (诊断日期________ 医院名称 ) 2.否

***f. 视网膜病变：***  1.是 (诊断日期________ 医院名称 ) 2.否

***g. 高血脂：*** 1.是 (诊断日期________ 医院名称 ) 2.否

**2.消化系统：**

***2.1 肝脏:***

***a.脂肪肝：***  1.是 (诊断日期________ 医院名称 ) 2.否

***b.病毒性肝炎*** 1.是 (诊断日期______ 医院名称

类型:乙肝 丙肝 其他类型 不确定 )

2.否

***c.肝硬化：*** 1.是 (诊断日期________ 医院名称 ) 2.否

***d.自身免疫性肝病***（如：自身免疫性肝炎、原发性胆汁性肝硬化、原发性硬化性胆管炎等）

1.是 (诊断日期________ 医院名称 ) 2.否

***e．其他肝脏疾病***（如肝脏良性肿瘤）：

_________________(诊断日期__________医院名称 )

***2.2 胆道：***

***a.胆囊炎：*** 1.是 (诊断日期________ 医院名称

是否手术治疗：是 否 )

2.否

***b.胆结石：*** 1.是 (诊断日期________ 医院名称

是否手术治疗：是 否 )

2.否

***c.胆囊息肉：*** 1.是 (诊断日期________ 医院名称

是否手术治疗：是 否 )

2.否

***d.其他胆道疾病：***

________________(诊断日期__________医院名称 )

***2.3 胰腺：***

***a.急性胰腺炎：*** 1.是 (诊断日期________ 医院名称

是否手术治疗：是 否 )

2.否

***b.慢性胰腺炎：***  1.是 (诊断日期________ 医院名称

是否手术治疗：是 否 )

2.否

***e.其他胰腺疾病***

_______________(诊断日期__________医院名称 )

***2.4 胃肠道：***

***a.慢性胃肠炎：*** 1.是 (诊断日期________ 医院名称 ) 2.否

***b.胃十二指肠溃疡：*** 1.是 (诊断日期________ 医院名称

是否手术治疗：是 否 )

2.否

***c.其他胃肠疾病：***

_______________(诊断日期__________医院名称 )

**3. 肾脏疾病**：

***a.肾结石：*** 1.是 (诊断日期________ 医院名称 ) 2.否

***b.肾囊肿：*** 1.是 (诊断日期________ 医院名称 ) 2.否

***c.慢性肾炎：*** 1.是 (诊断日期________ 医院名称 ) 2.否

***d.肾病综合症：*** 1.是 (诊断日期________ 医院名称 ) 2.否

***e. 其他肾脏疾病：***

_______________(诊断日期__________医院名称 )

**4. 呼吸系统：**

***a.慢性支气管炎：***1.是 (诊断日期________ 医院名称 ) 2.否

***b.肺气肿：*** 1.是 (诊断日期________ 医院名称 ) 2.否

***c.其他呼吸系统疾病：***

　 _______________（诊断日期_____ __医院名称 )

***d.过去12个月里，夜间睡眠时是否有打鼾？*** 1.经常 2.偶尔 3.从不 99.不清楚

**5. *骨折：*** 1.是

部位1：____________ 年龄1：___ ____岁 原因1：

部位2：____________ 年龄2：_________岁 原因2：

部位3：____________ 年龄3： ______岁 原因3：

2.否

**6．是否得过血吸虫病？**

1.是 2.否

－如果否，请进入**7 其他疾病**。

－如果是，请继续以下问题。

请注明感染血吸虫病时年龄： 岁

请注明接受过以下哪些治疗： 1. 抗血吸虫病药物 2. 切脾治疗 3.不清楚

请注明是否曾经诊断过血吸虫肝病：1. 是 2. 否 3.不清楚

**7.其他疾病 （除糖尿病）**

___________________(诊断日期__________医院名称 )

___________________(诊断日期__________医院名称 )

**问题3：家族史－――糖尿病和肿瘤**

**在您的血缘亲属中（包括已过世的）,是否有人患有（或患过）恶性肿瘤或糖尿病：**

1.是 2.否

－如果否，请进入问题4。

－如果是，包括：（**可多选**，若有患病，则在“是否患病”处圈选“1”；如不存在患病，则在“是否患病”处圈选“2”。若其中患病者为儿子、女儿、兄弟、姐妹，请详细指出各选项的患病者个数；若患病者为父母亲、祖父母、外祖父母，则患病人数此项不填）

|  | 肿瘤家族史 | | | | | | 糖尿病家族史 | | |
| --- | --- | --- | --- | --- | --- | --- | --- | --- | --- |
|  | 是否患病 | | 患病人数 | 部位1 | 部位2 | 部位3 | 是否患病 | | 患病人数 |
| 父亲 | 1.是 | 2.否 |  |  |  |  | 1.是 | 2.否 |  |
| 母亲 | 1.是 | 2.否 |  |  |  |  | 1.是 | 2.否 |  |
| 儿子 | 1.是 | 2.否 |  |  |  |  | 1.是 | 2.否 |  |
| 女儿 | 1.是 | 2.否 |  |  |  |  | 1.是 | 2.否 |  |
| 兄弟 | 1.是 | 2.否 |  |  |  |  | 1.是 | 2.否 |  |
| 姐妹 | 1.是 | 2.否 |  |  |  |  | 1.是 | 2.否 |  |
| 祖父 | 1.是 | 2.否 |  |  |  |  | 1.是 | 2.否 |  |
| 祖母 | 1.是 | 2.否 |  |  |  |  | 1.是 | 2.否 |  |
| 外祖父 | 1.是 | 2.否 |  |  |  |  | 1.是 | 2.否 |  |
| 外祖母 | 1.是 | 2.否 |  |  |  |  | 1.是 | 2.否 |  |

**问题4：您诊断糖尿病至今是否曾经接受过降糖治疗？**

1.是 2.否

－如果否：请进入问题5。

－如果是：请注明哪种类型：1.口服药物 2.胰岛素 3.口服药物+胰岛素

**您一年内服用过降糖药物或注射过胰岛素吗？**

1.是 2.否

－如果否：请进入问题5。

－如果是：请详细描述您服用药物的名称、每日剂量、开始服用的日期及累计服用时程。

|  | 药物类别 | 药物名称 | 药物剂量（/天） | 开始日期（年/月） | 服用时程(月) |
| --- | --- | --- | --- | --- | --- |
| 1 | 磺脲类促泌剂 |  |  |  |  |
| 2 | 非磺脲类促泌剂 |  |  |  |  |
| 3 | 双胍类 |  |  |  |  |
| 4 | 糖苷酶抑制剂 |  |  |  |  |
| 5 | 噻唑烷二酮 |  |  |  |  |
| 6 | 普通胰岛素 |  |  |  |  |
| 7 | 甘精胰岛素 |  |  |  |  |
| 8 | GLP1类似物 |  |  |  |  |
| 9 | DPP4抑制剂 |  |  |  |  |
| 10 | 中药 |  |  |  |  |
| 11 | 其它 |  |  |  |  |

| **表一 糖尿病常用药物简表** | |
| --- | --- |
| **磺脲类促泌剂** | - 格列本脲（优降糖） - 格列齐特（达美康） - 格列吡嗪（美吡达；优哒灵；瑞易宁；秦苏；迪沙；灭特尼） - 格列喹酮（糖适平；捷适） - 格列美脲（亚莫利；安多美；瑞平；伊瑞；万苏平） - 甲苯磺丁脲（D860片） |
| **非磺脲类促泌剂** | - 瑞格列奈（诺和龙；孚来迪） - 那格列奈（唐力） |
| **双胍类** | - 二甲双胍（格华止；美迪康；美福明；甲福明；迪化糖啶；文达敏^注^） - 苯乙双胍（降糖灵） |
| **糖苷酶抑制剂** | - 阿卡波糖（拜唐苹；卡博平） - 伏格列波糖（倍欣） |
| **噻唑烷二酮** | - 罗格列酮（文迪雅；文达敏^注^） - 吡格列酮（卡司平；艾汀；艾可拓） |
| **普通胰岛素**^注^ | - 门冬胰岛素（诺和锐） - 赖脯胰岛素（优泌乐） - 人胰岛素（诺和灵；优泌林；甘舒霖） - 动物胰岛素（中性胰岛素注射液；万苏林） |
| **甘精胰岛素** | - 来得时；长秀霖；诺和平 |
| **GLP1类似物** | - 利拉鲁肽（Liraglutide） |
| **DPP4抑制剂** | - 维达利停（Vildagliptin） - 西他列汀（Januvia） |
| **中药** | - 消渴丸 - 金芪降糖片 |

注：文达敏为二甲双胍和罗格列酮复合剂型；胰岛素泵使用者记录为普通胰岛素组；

**问题5：您近二周（包括今天）服用过其他药物吗？(除降糖药物以外)**

1.是 2.否

－如果否：请进入问题6。

－如果是：请详细描述您服用药物的名称、每日剂量、开始服用的日期及累计服用时程。

|  | 药物类别 | 药物名称 | 药物剂量（/天） | 开始日期（年/月） | 服用时程(月) |
| --- | --- | --- | --- | --- | --- |
| 1 |  |  |  |  |  |
| 2 |  |  |  |  |  |
| 3 |  |  |  |  |  |
| 4 |  |  |  |  |  |
| 5 |  |  |  |  |  |

**问题6：吸烟情况（每天吸烟的定义:至少半年以上每天吸烟1支或每周7支）**

**主动吸烟情况：**

**6.1** 请问您现在吸烟吗？

1.现在不

2.偶尔（不是每天）

3.是，每天或几乎每天( **请进入 6.4** )

**6.2** 请问您过去的吸烟习惯：

1.从不吸烟（ **请进入问题 6.8** ）

2.过去偶尔吸烟（少于每天一次） （ **请进入问题 6.8** ）

3.过去习惯每天吸烟

**6.3** 请问您戒烟多少年了? 年

**6.4** 您现在或在戒烟前，吸烟的量是多少？

香烟 支/天

雪茄 支/天

手卷烟 两/月

**6.5** 请问您吸烟时吸入烟雾吗？ 1.是 2.否

**6.6** 您大约从几岁开始习惯性每天吸烟？ 岁

**6.7** 您习惯性每天吸烟多少年了？ 年

**被动吸烟情况：**

**6.8** 您18岁之前家里是否有人吸烟？ 1.是 2.否

**6.9** 您家里是否有人吸烟？

1.是

您目前家里有 人吸烟

每天家人在你面前总共吸烟 支

这种情况存在 年

每周平均 天你会暴露在这种烟雾环境中

每天家里在烟雾环境中暴露 分钟

2.否

**6.10** 是否有同事在您面前吸烟（可以让你感觉吸入烟雾）？

1.是

工作环境共有 人吸烟

每天你周围同事在你面前的总吸烟量约有 支

这种情况存在 年

每周平均 天暴露在这种烟雾环境中

每天在这种烟雾工作环境中暴露 分钟

2.否

**问题7：饮酒情况 （询问过去的12个月中，饮用包括啤酒、白酒、黄酒、葡萄酒以及其他的含酒精饮料的情况。经常饮酒的定义:至少半年以上，每周饮酒至少一次）**

**7.1** 请问您现在饮酒吗？

1.现在不

2.偶尔（不是每周）

3.是，每周或几乎每周( **请进入7.4** )

**7.2** 请问您过去的饮酒习惯：

1.从不饮酒（ **请进入问题8** ）

2.过去偶尔饮酒(少于每周一次) （ **请进入问题8** ）

3.过去习惯每周饮酒

**7.3** 请问您戒酒多少年了？ 年

**7.4** 请问您现在或在戒酒前，饮酒的量和频率是怎样的？

白酒（威士忌）： 两／次 ； 次/周

黄酒（米酒）： 两／次 ； 次/周

葡萄酒： 两／次 ； 次/周

啤酒（750ml/瓶）： 瓶／次 ； 次/周

其他种类酒 两／次 ； 次/周

**7.5** 您习惯性饮酒多少年了？ 年

**7.6** 您近一年来是否有一次大量饮酒或醉酒的经历（具体如下）：

A.只饮白酒,每次3两或3两以上：

1. 是 每次 两

2. 否

B.只饮黄酒,每次5两或5两以上：

1. 是 每次 两

2. 否

C.只饮葡萄酒,每次5两或5两以上：

1. 是 每次 两

2. 否

D.只饮啤酒（750ml/瓶）,每次2瓶或2瓶以上：

1. 是 每次 瓶

2. 否

E.每次饮两种及两种以上酒：

1. 是 每次： 两/白酒

两/黄酒

两/红酒

瓶/啤酒（750ml/瓶）

2. 否

**7.7** 您近一年来大量饮酒或醉酒的次数： 次/月

**问题8：饮茶情况（茶饮料除外）**

**8.1**下面哪种情况最能反映您过去一年的饮茶情况？

1.从不或几乎从不( **请进入问题9** )

2.偶饮（即每月最多1—3次，每周少于一次）( **请进入问题9**)

3.过去常饮茶，但现在不饮茶了

4.现在经常饮茶（每周最少有一天饮）（ **请进入8.3** ）

**8.2** 您停止饮茶有几年了？ 年（如少于一年则填“0”）

**8.3** 请问您现在或停止饮茶前，饮茶的量和频率是怎样的？（按150毫升容量的杯计算，请选您平时最常饮用的一种茶回答）

绿茶类/茉莉花茶／龙井／白茶 杯/天 天/周

乌龙茶类（铁观音、水仙等） 杯/天 天/周

红茶类 （包括砖茶，普洱） 杯/天 天/周

其他 (请注明， ) 杯/天 天/周

**8.4** 在过去一年里，您本人平均每月饮用的茶叶总量大约是多少？

两／月

**8.5** 您大约从几岁开始习惯性的饮茶（每周最少有一天饮）？ 岁

**问题9：职业与环境危险因素**

| 9.1 | 过去一年内，您是否接触过油漆行业？ | 1.经常 2.偶尔 3.从不 99.不清楚 |
| --- | --- | --- |
| 9.2 | 过去一年内，您是否接触过放射线？ | 1.经常 2.偶尔 3.从不 99.不清楚 |
| 9.3 | 过去一年内，您是否接触过化学物质？ | 1.经常 2.偶尔 3.从不 99.不清楚 |
| 9.4 | 过去一年内，您家中或工作单位是否进行过装修？ | 1.是 2.否 99.不清楚 |
| 9.5 | 过去一年内，您的居住地附近是否有化工厂或核电站？ | 1.是 2.否 99.不清楚 |

**问题10：体重变化**

| **10.1** | 今天之前您最近一次测量体重的时间是？ | | | | | | | | 1. 从未量过  2. 1个月内  3. 6个月内  4. 6-12 个月  5. 12个月前  99.记不清 | |
| --- | --- | --- | --- | --- | --- | --- | --- | --- | --- | --- |
| **10.2** | 同一年前相比，你的体重是 | | | | | | | | 1.减轻  2.增加  3.基本不变  99. 不清楚 | |
| **10.3** | 过去12 个月里，您是否采取过措施改变体重？ | | | | | | | | 1.减重  2.增重  3.未尝试改变 | |
| **10.4** | 如果您正在尝试减轻体重，你使用的方法有哪些？（可多选） | | | | | | | | 1.控制饮食  2.锻炼  3.药物  99.其他（请注明） | |
| **10.5** | 请您回忆以下不同阶段的体重 | | | | | | | | 仅针对女性 | |
| **时间** | 出生时 | 20岁 | 30岁 | 40岁 | 50岁 | 60岁 | 70岁 | >70岁 | 生育前 | 生育后 |
| **（Kg）** |  |  |  |  |  |  |  |  |  |  |

**问题11：情绪情况**

| 11．1 | 最近2周里，您是否觉得，做什么事情  都没有兴趣，没有意思？ | 1 没有  2 有几天，但不多  3 超过一周  4 几乎每天都是  99 不清楚  97 拒绝回答 |
| --- | --- | --- |
| 11．2 | 最近2周里，您是否觉得心情低落，沮丧，或者没有希望？ | 1 没有  2 有几天，但不多  3 超过一周  4 几乎每天都是  99 不清楚  97 拒绝回答 |
| 11．3 | 最近2周里，您是否睡不着，睡不踏实，  或者睡得太多？ | 1 没有  2 有几天，但不多  3 超过一周  4 几乎每天都是  99 不清楚  97 拒绝回答 |
| 11．4 | 最近2周里，您是否觉得疲惫，没劲？ | 1 没有  2 有几天，但不多  3 超过一周  4 几乎每天都是  99 不清楚  97 拒绝回答 |
| 11．5 | 最近2周里，您是否没有胃口，或者吃  得太多？ | 1 没有  2 有几天，但不多  3 超过一周  4 几乎每天都是  99 不清楚  97 拒绝回答 |
| 11．6 | 最近2周里，您是否对自己不满，或者  觉得自己是个失败者，或者让您或您的  家人感到失望？ | 1 没有  2 有几天，但不多  3 超过一周  4 几乎每天都是  99 不清楚  97 拒绝回答 |
| 11．7 | 最近2周里，您是否觉得，无法专心做  事，比如读书、看报或者看电视？ | 1 没有  2 有几天，但不多  3 超过一周  4 几乎每天都是  99 不清楚  97 拒绝回答 |
| 11．8 | 最近2周里，您是否觉得，自己行动或  说话变得迟缓，以致引起别人的注意，  或者相反，坐立不安，心情烦躁，比平  时更易到处走动？ | 1 没有  2 有几天，但不多  3 超过一周  4 几乎每天都是  99 不清楚  97 拒绝回答 |
| 11．9 | 最近2周里，您是否有过轻生的念头，  或者伤害自己的想法？ | 1 没有  2 有几天，但不多  3 超过一周  4 几乎每天都是  99 不清楚  97 拒绝回答 |
| 11.10 | 如果您有上面所问及的问题（11.1-11.9），他们对您平时工作、家庭生活，与人相处有没有影响？ | 1 没有影响  2 有些影响  3 很有影响  4 极大影响  99 不清楚  97 拒绝回答 |
| 11．11 | 过去12 个月里，您和家人（如父母、配偶、子女）之间的关系如何？ | 1 非常好  2 好  3 一般  4 差  5 非常差  6 独居无家人  97 拒绝回答 |
| 11．12 | 过去12 个月里，您是否遇到对自己打击比较大的事情？ | 1 有  2 没有  97 拒绝回答 |
| 11．13 | 过去12 个月里，总的来说，您对自己的生活是否满意？ | 1 很满意  2 满意  3 一般  4 不满意  5 很不满意  97 拒绝回答 |

**问题12：饮食情况**

| 12.1 | | 过去12个月内，您通常1天吃几顿饭？ | | | 顿 | | | | | |
| --- | --- | --- | --- | --- | --- | --- | --- | --- | --- | --- |
|  |  | 过去12个月里，您通常一周内吃早餐的天数？ | | | | | | 天 | | |
|  |  | 过去12个月里，您通常一周内吃午餐的天数？ | | | | | | 天 | | |
|  |  | 过去12个月里，您通常一周内吃晚餐的天数？ | | | | | | 天 | | |
|  |  | 过去12个月里，您通常一周内加用夜宵的天数？ | | | | | | 天 | | |
|  | | | | | 就餐地点 | | | | | |
|  |  |  |  |  | a家 | | b食堂 | | c餐馆 | |
| 12.2 | | 过去12 个月里，您通常一周在不同就餐地点吃早餐的天数？ | | | 天 | | 天 | | 天 | |
| 12.3 | | 过去12 个月里，您通常一周在不同就餐地点吃中餐的天数？ | | | 天 | | 天 | | 天 | |
| 12.4 | | 过去12 个月里，您通常一周在不同就餐地点吃晚餐的天数？ | | | 天 | | 天 | | 天 | |
| 请回忆过去12个月里通常情况下，您是否吃过下列食物，并估计各类食物的食用频率和食用量。 | | | | | | | | | | |
|  |  | | a 是否食用 | b食用频率（只填其中1项） | | | | | | 平均每次食用量 |
|  |  | | 1 是，  2 否 | b 1  次数/天 | b 2  次数/周 | b 3  次数/月 | | b4  次数/年 | | 两 |
| 12.5 | 米、面、杂粮等粮谷类食物(按生重记录) | |  |  |  |  | |  | | 两 |
| 12.6 | 薯类（土豆/芋头/红薯） | |  |  |  |  | |  | | 两 |
| 12.7 | 猪肉（按生重记录) | |  |  |  |  | |  | | 两 |
| 12.8 | 牛、羊等畜肉  (按生重记录) | |  |  |  |  | |  | | 两 |
| 12.9 | 鸡鸭鹅等禽肉 | |  |  |  |  | |  | | 两 |
| 12.10 | 水产品  (鱼虾类，按生重计) | |  |  |  |  | |  | | 两 |
| 12.11 | 新鲜蔬菜 | |  |  |  |  | |  | | 两 |
| 12.12 | 新鲜水果 | |  |  |  |  | |  | | 两 |
| 12.13 | 鲜榨蔬果汁(250ml/杯) | |  |  |  |  | |  | | 杯 |
| 12.14 | 蛋类(以鸡蛋计) | |  |  |  |  | |  | | 个 |
| 12.15 | 奶制品(以鲜奶计) | |  |  |  |  | |  | | 两 |
| 12.16 | 豆制品(以豆腐计) | |  |  |  |  | |  | | 两 |
| 12.17 | 油炸食品(油条、油饼等) | |  |  |  |  | |  | | 两 |
| 12.18 | 果蔬汁/果味饮料(250ml/杯) | |  |  |  |  | |  | | 杯 |
| 12.19 | 碳酸饮料(250ml/杯) | |  |  |  |  | |  | | 杯 |
| 12.20 | 糕点 | |  |  |  |  | |  | | 两 |
| 12.21 | 咸菜 | |  |  |  |  | |  | | 不用“填写” |
| 12.22 | 泡菜 | |  |  |  |  | |  | |  |
| 12.23 | 腐乳 | |  |  |  |  | |  | |  |
| 12.24 | 咖啡 | |  |  |  |  | |  | |  |
| 12.25 | 动物内脏 | |  |  |  |  | |  | |  |
| 12.26 | 营养素补充剂( 如维生素、矿物质等) | |  |  |  |  | |  | |  |

表**2** 食物折算表


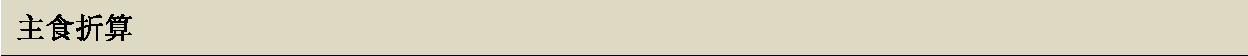


| 食物名称 | 单位 | 相当于生米或生面的重量克 | 两 |
| --- | --- | --- | --- |
| 大米饭 | 1 小标准碗（碗直径12cm） | 75 | 1.5 |
|  | 1 大标准碗（碗直径16cm） | 150 | 3.0 |
| 大米粥 | 1 小标准碗（碗直径12cm） | 30 | 0.6 |
|  | 1 大标准碗（碗直径16cm） | 50 | 1.0 |
| 馒头 | 1 个（需根据大小折算） | 100 | 2.0 |


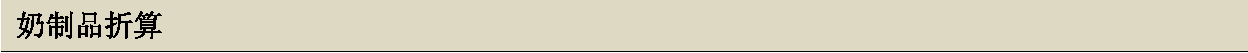


| 奶制品名称 | 等于2 两（100 克）鲜牛奶的重量 |
| --- | --- |
| 鲜牛奶（羊奶） | 2.0 两（100克） |
| 酸奶 | 2.0 两（100克） |
| 奶粉 | 0.3 两（15克）（约1 勺半） |
| 奶酪 | 0.2 两（10克） |


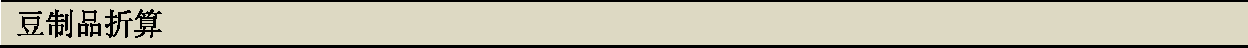


| 豆制品名称 | 等于2 两（100 克）北豆腐的重量 |
| --- | --- |
| 北豆腐 | 2.0 两 （100 克） |
| 内酯豆腐 | 4.8 两 （240 克） |
| 豆腐干 | 1.5 两 （75 克） |
| 豆腐丝 | 1.0 两 （55 克） |
| 腐竹 | 0.5 两 (25 克) |
| 豆浆 | 10.0 两（500 克） |

**问题13：过去七天的体力活动及睡眠情况**

**13.1** 请问您是否还在工作（全职或兼职）

1.是； 2.否；

－如果是：请注明工种：________________；

工作时：1.不太活动（办公室等） 每周 天， 每天 小时

2.轻度活动（流水线工作等） 每周 天， 每天 小时

3.中度活动（安装工、搬运工等） 每周 天， 每天 小时

4.重度活动（炼钢、农业、铸造等） 每周 天， 每天 小时

**13.2** **请提供您在过去的七天里面，工作以外业余时间剧烈体力活动的情况**（剧烈活动是指令您觉得呼吸吃力的很多活动，例如举重物,打篮球，游泳，跑步等。请您只计算每次持续十分钟或十分钟以上的活动)。

1.有 每周 天， 每天 小时

2.无 每周0天

**13.3 请提供您在过去的七天里面，工作以外业余时间中等强度体力活动的情况**（中等强度的体力活动指令您呼吸稍比正常吃力的活动，例如：缓步跑，打乒乓球，耍太极等，但不包括步行。请您只计算每次持续十分钟或十分钟以上的活动)。

1.有 每周 天， 每天 小时

2.无 每周0天

**13.4请提供您在过去的七天里面，步行活动的情况**（包括工作及业余时间任何形式的步行。请您只计算每次持续十分钟或十分钟以上的步行）

1.有 每周 天， 每天 小时

2.无 每周0天

**13.5请回忆在过去12个月里通常情况下，您是否进行过以下活动，并估计各类活动的频率和时间。**

|  | a是否活动 | b频率（只填其中1项） | | | | 平均每次活动时间 |
| --- | --- | --- | --- | --- | --- | --- |
|  | 1 是，  2 否 | b 1  次数/天 | b 2  次数/周 | b 3  次数/月 | b4  次数/年 |  |
| **瑜伽** |  |  |  |  |  | \| \| \|小时\| \| \|分钟 |
| **太极** |  |  |  |  |  | \| \| \|小时\| \| \|分钟 |
| **健美操** |  |  |  |  |  | \| \| \|小时\| \| \|分钟 |
| **舞蹈** |  |  |  |  |  | \| \| \|小时\| \| \|分钟 |
| **健身器材** |  |  |  |  |  | \| \| \|小时\| \| \|分钟 |
| **其它** |  |  |  |  |  | \| \| \|小时\| \| \|分钟 |
| **其它** |  |  |  |  |  | \| \| \|小时\| \| \|分钟 |

**13.6 请提供您在过去一周，坐着的情况**（包含工作及休闲时的坐着）

**周一到周五：**

1.有 每周 天，每天 小时

2.无

**周六和周日：**

1.有 每周 天，每天 小时

2.无

**13.7 在过去的7天里，您每天睡眠情况**

1. 睡眠感觉良好: 每天中午睡 小时 分钟，晚上从 : 睡到 :

2. 睡眠感觉不好: 每天中午睡 小时 分钟，晚上从 : 睡到 :

3. 安眠药助眠: 每天中午睡 小时 分钟，晚上从 : 睡到 :

**13.8 过去12个月里，通常情况下您平均每天看电视的时间为多少？**

| | |小时| | |分钟

**13.9 过去12个月里，通常情况下您平均每天使用电脑的时间为多少？**

| | |小时| | |分钟

**13.10 过去12个月里，通常情况下您平均每天用于阅读（纸质书籍）的时间为多少？**

| | |小时| | |分钟

**13.11 过去12个月里，通常情况下您平均每天用于玩电子游戏（不包括电脑游戏）的时间为多少？**

| | |小时| | |分钟

**13.12 您平时使用手机吗？**

1.经常 请问您已经使用手机多少年了？ 年

2.偶尔

3.从不

**问题14：请提供有关您生育的信息。**

**14.1 男性：**

●您生育了几个孩子？ 个 男： 个；女： 个

●您生育的孩子中是否有巨大胎儿（出生体重> 4 kg）

1.是 个 男： 个；女： 个 2.否

**14.2女性：**

●您怀孕次数？ 次

●您流产次数？ 次 其中自然流产 次 人工流产 __次

●您生育了个孩子？ 个 男： 个；女： 个

●您生育的孩子中是否有巨大胎儿（出生体重> 4 kg）

1.是 个 男： 个；女： 个 2.否

●您怀孕期间是否有过妊娠高血压：1.是 2.否

●您怀孕期间是否有过妊娠高血糖：1.是 2.否

●您怀孕期间是否有过宫外孕： 1.是 __次 2.否

●您是否对子女母乳喂养？

1. 是 母乳喂养时间：平均 ___月/每个子女

2. 否

**问题15：（只针对女性询问）请提供有关您月经的信息。**

**15.1** 请问您何时月经初潮 _ 年 月( 岁)

**15.2** 请问您还有月经吗？

1.是，请问您最近一次月经的时间 _ 年 月（**请进入15.5**）

2.否

**15.3** 请说明您何时完全绝经 _ 年 月( 岁)

**15.4** 您是自然绝经的吗？（如果是自然绝经，尽管后来您做了涉及生殖器官的手术，该问题请回答“是”）

1.是

2.否。请描述您做的手术，说明手术日期（年/月）、原因及实施手术的医院名称。

| 手术名称 | 手术日期（年/月） | 手术原因 | 实施手术的医院名称 |
| --- | --- | --- | --- |
|  |  |  |  |
|  |  |  |  |

**15.5** 您曾经服过避孕药或使用性激素替代治疗吗？

1.是。请描述您服用药物的名称、每日剂量、开始服用的时间及累计服用时程。

| 药物名称 | 药物剂量（/天） | 开始日期（年/月） | 服用时程(月) |
| --- | --- | --- | --- |
|  |  |  |  |
|  |  |  |  |

2.否

**第三部分 临床检查**

**1．血压测量（欧姆龙）** 调查员姓名：

您今天服用降压药了吗？ 1.是 2.否

您是否静坐了5分钟？ 1.是 2.否（请您静坐5分钟后开始测量）

测量时间 ： （小时/分） 测量的上臂 1 =右, 2 =左

收缩压1 /舒张压1 mmHg 脉率1 /分

**（等候1分钟）**

收缩压2 /舒张压2 mmHg 脉率2 /分

**（等候1分钟）**

收缩压3 /舒张压3 mmHg 脉率3 /分

**2．临床测量**

身高（精确到0.1cm） . cm 体重（精确到0.1kg） . kg

腰围（精确到0.1cm） . cm 臀围（精确到0.1cm） . cm

**3. 心电图（EKG）：** 1. 已完成 2. 未完成 调查员姓名：

**4. 化验检查**

**血液标本收集：** 是否空腹：1 = 是，2 = 否

最后一餐日期 月 日

最后一餐时间 ： （小时/分）

**0分钟抽血**  1 = 是，2 = 否

**2小时抽血**  1 = 是，2 = 否

**今天是否服用降糖药或注射胰岛素** 1 = 是，2 = 否；

**服药（胰岛素）时间：** ： （小时/分）

**第四部分 辅助检查**

**1、糖尿病心脑血管并发症**

**1．1超声检查——颈动脉内中膜厚度（IMT）测定：**

左侧颈动脉内中膜厚度 毫米； 右侧 颈动脉内中膜厚度 毫米。

是否有斑块：1） 有 斑块位置 a.左侧 斑块性质 1)均质性 斑块个数 个

2)非均质性 斑块个数 个

b.右侧 斑块性质 1)均质性 斑块个数 个

2)非均质性 斑块个数 个

2） 无

调查员姓名：

**1．2血管功能检查（PWV、ABI、TBI）：** 1. 已完成 2. 未完成 调查员姓名：

**2、糖尿病视网膜病变**

**眼底摄片检查：** 1. 已完成 2. 未完成 调查员姓名：

**3、糖尿病肾脏病变**

**尿微量白蛋白/肌酐：** 1. 已收集待测尿样 2. 未收集待测尿样 调查员姓名：

**4、骨质疏松**

**骨密度检查：** 1. 已完成 2. 未完成 调查员姓名：

请圈选以下检查结果前的字母：　a．骨密度正常

b．骨密度下降

c．骨质疏松
